# A quantitative content analysis of Chinese policies on fertility-friendly hospital development: a three-dimensional framework

**DOI:** 10.3389/fpubh.2026.1953541

**Published:** 2026-09-04

**Authors:** Zichao Zhang, Zhensheng Guo, Ruixiang Yang, Qiaoyang Zhang, Guiyun Ma

**Affiliations:** 1 School of Nursing, Chengde Medical University, Chengde, China; 2 Graduate School of Hebei North University, Zhangjiakou, China; 3 Department of Nursing, Affiliated Hospital of Chengde Medical College, Chengde, China

**Keywords:** fertility-friendly hospitals, policy objectives, policy text analysis, policy tools, three-dimensional analysis

## Abstract

**Background:**

This study examined the characteristics of Chinese policy documents on fertility-friendly hospital development using a policy-tool lens, identified patterns of policy emphasis and areas receiving comparatively less attention, and provided evidence for further policy improvement.

**Methods:**

A three-dimensional analytical framework encompassing policy tools, stakeholders, and policy objectives was developed and applied to a quantitative content analysis of 52 national- and provincial-level policy documents on fertility-friendly hospital development issued between April 2016 and February 2026.

**Results:**

The distribution of policy tools was uneven, with supply-side and environmental tools accounting for 53.29 and 34.69% of policy-tool coded references, respectively, whereas demand-side tools accounted for 12.02%. Maternity care institutions were the most frequently represented stakeholder group, accounting for 42.12% of stakeholder-related coded references. Among the policy objectives, the promotion of institutional friendliness and the establishment of supportive mechanisms received the greatest emphasis, accounting for 36.44% of policy-objective coded references.

**Conclusion:**

The included policies were characterized by greater emphasis on supply-side and environmental tools, maternity care institutions, and selected policy objectives, whereas demand-side tools and broader stakeholder participation received comparatively less attention. Future policy development may consider broadening the use of demand-side tools, increasing explicit policy attention to primary healthcare institutions and social organizations, and promoting broader stakeholder participation. Such changes may support a more comprehensive, multidimensional, and collaborative governance framework spanning the continuum of care.

## 1 Introduction

Population dynamics are closely linked to national development and represent a long-term strategic issue for China (1). China is currently undergoing a profound demographic transition characterized by accelerated population aging and declining fertility rates, posing new challenges to long-term and balanced population development (2). Against this backdrop, optimizing the provision of maternity healthcare services has become an important policy priority for reducing concerns related to childbearing and supporting fertility decisions. In 2024, four national departments, including the National Health Commission, jointly issued the “Opinions on Promoting the Development of Fertility-Friendly Hospitals,” defining fertility-friendly hospitals as healthcare institutions that integrate fertility-friendly principles into all aspects of healthcare services and operational management (3). The document calls for systematic improvements in areas including institutional mechanisms, physical environments, continuous services, service models, and clinical processes, aiming to enhance public access to and satisfaction with reproductive healthcare services.

Internationally, efforts to improve maternal and newborn care quality have been guided by frameworks emphasizing woman-centered care, respectful maternity care, and positive childbirth experiences (4, 5). For example, the WHO/UNICEF Baby-Friendly Hospital Initiative emphasizes the provision of supportive care environments for mothers and newborns (4), while WHO recommendations on intrapartum care for a positive childbirth experience further highlight respectful care, effective communication, and continuous support during childbirth (5). China’s fertility-friendly hospital initiative is consistent with these internationally recognized principles. At the same time, China’s fertility-friendly hospital initiative integrates demographic development needs with fertility-supportive policy objectives and embeds healthcare service optimization within broader population development strategies, representing a context-specific pathway for improving fertility-related healthcare services. Therefore, integrating fertility-friendly principles into healthcare institution development represents an important component of strengthening China’s fertility support policy system.

In recent years, China has introduced a series of policies to support childbearing, gradually extending fertility-friendly principles to healthcare institutions. In June 2021, the State Council issued the “Decision on Optimizing the Fertility Policy to Promote Long-Term and Balanced Population Development,” which called for better care for pregnant and postpartum women, an improved childbirth experience, enhanced hospital-based maternal and infant health services, and stronger safeguards for maternal and infant safety (6). In July 2022, the National Health Commission and 17 other departments issued the “Guiding Opinions on Further Improving and Implementing Measures to Support Proactive Childbearing.” The document further strengthened services for healthy conception and childbirth by promoting high-quality obstetric care, expanding pilot programs for labor analgesia, and standardizing the provision of assisted reproductive technologies (7). In October 2024, the General Office of the State Council issued the “Several Measures to Accelerate the Improvement of the Fertility Support Policy System and Promote the Development of a Fertility-Friendly Society.” The policy emphasized reducing the costs associated with childbearing and child-rearing and creating a more supportive social environment (8). Building on these policy initiatives, the National Health Commission and three other departments jointly issued the “Opinions on Promoting the Development of Fertility-Friendly Hospitals” in December 2024. The policy aims to support women throughout conception, pregnancy, childbirth, and the postpartum period, while improving the quality of maternal and child health services (3).

Although policy experience in this area has gradually accumulated, few studies have systematically reviewed or examined policy documents concerning the development of fertility-friendly hospitals. Accordingly, this study sought to address three interrelated questions: (1) what policy tools are used to promote fertility-friendly hospital development; (2) which stakeholders are represented in policy formulation and implementation; and (3) what policy objectives are emphasized and how policy tools, stakeholders, and policy objectives are aligned. To address these questions, this study analyzed national- and provincial-level policy documents on fertility-friendly hospital development issued between April 2016 and February 2026 using a three-dimensional analytical framework encompassing policy tools, stakeholders, and policy objectives. The findings may provide evidence to support the further development of fertility-friendly hospitals and the promotion of maternal and infant health.

## 2 Materials and methods

Using a three-dimensional framework encompassing policy tools, stakeholders, and policy objectives, this study employed quantitative content analysis to examine national- and provincial-level policies related to fertility-friendly hospital development in China. First, policy documents related to fertility-friendly hospital development were identified through official government websites and policy databases. Second, content analysis was conducted to systematically code and quantify policy content across the three dimensions. Given that administrative levels may shape policy priorities and implementation contexts, an exploratory subgroup analysis was conducted by stratifying policy documents into national- and provincial-level groups to compare the distribution patterns of policies across the three dimensions. Finally, based on the findings, recommendations were proposed to strengthen China’s policy framework for fertility-friendly hospital development by optimizing the policy tool mix, enhancing stakeholder participation, and refining policy objectives. An exploratory word-frequency analysis was also conducted using NVivo 15, with the corresponding methods and results provided in Supplementary file S1.

### 2.1 Policy text selection

To ensure the authority and comprehensiveness of the policy corpus, we searched official websites of national government authorities, including the State Council, the National Health Commission, the National Development and Reform Commission, and the National Healthcare Security Administration, as well as official websites of provincial-level governments and relevant administrative departments. The Peking University Law Database was additionally searched. CNKI was used as a supplementary source to cross-check policy titles and identify potentially relevant documents, thereby reducing the risk of omission.

The original Chinese search terms included “生育友好” (“fertility-friendly”), “母婴友好” (“maternal- and infant-friendly”), and “助产医院” (“maternity hospital”). Policy documents identified through supplementary searches were verified against official government websites or authoritative policy databases before being included in the analysis. The search covered policy documents issued from April 2016 to February 2026, with the final search conducted on April 28, 2026. The identification and selection of policy documents were reported in accordance with the READ approach for document analysis in health policy research (9).

Inclusion criteria: (1) documents directly related to fertility-friendly hospital development and containing substantive policy objectives or implementation measures; (2) documents issued by national authorities, provincial-level governments, or relevant administrative departments; and (3) formally issued policy documents, such as plans, notices, opinions, regulations, or decisions. Exclusion criteria: (1) documents only marginally related to fertility-friendly hospital development or lacking substantive policy objectives or implementation measures; (2) policy interpretations, public announcements, approval documents, and other non-policy materials; (3) duplicate documents; and (4) policy documents no longer valid at the retrieval cutoff date.

**Figure 1:**
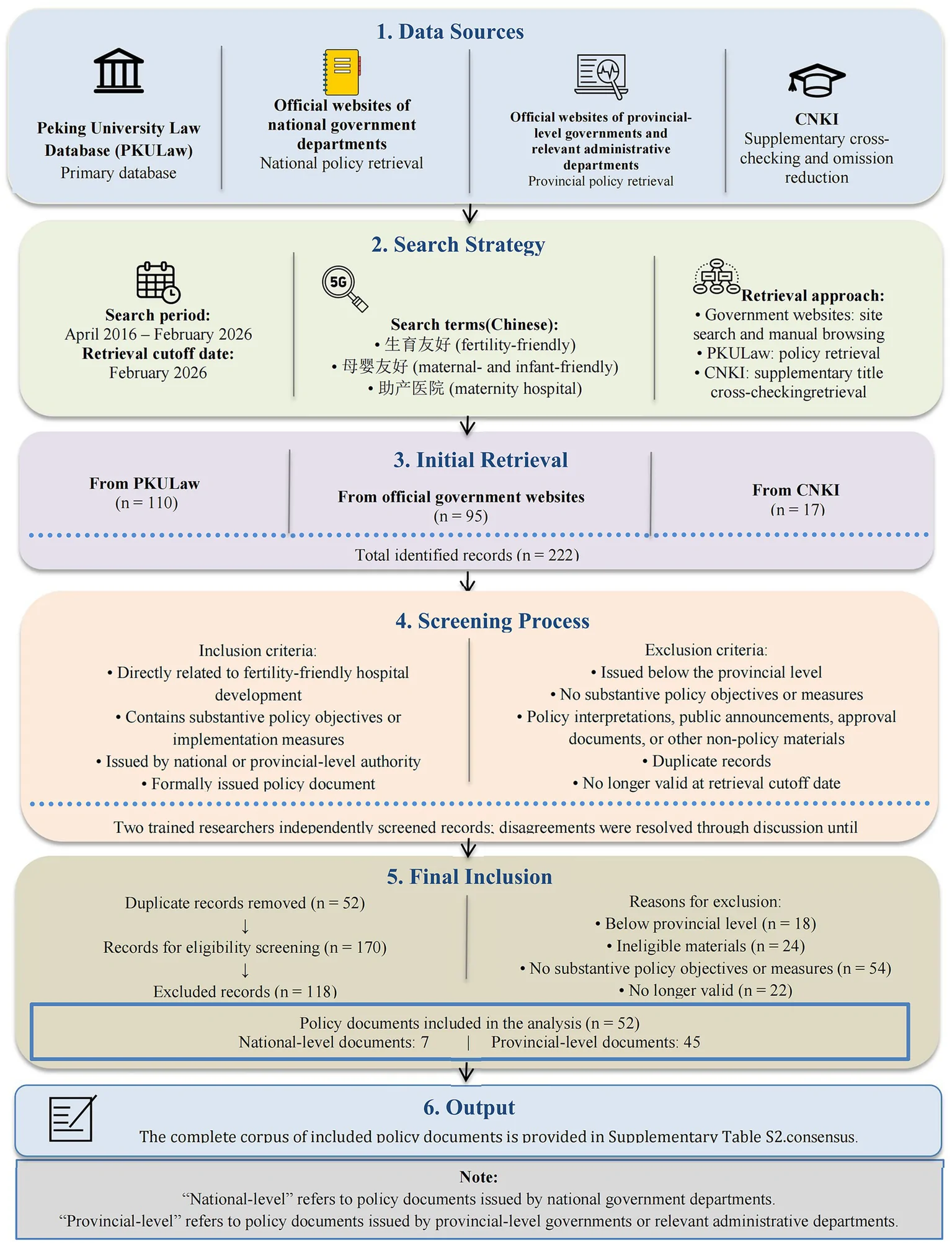
Retrieval and screening process of policy documents.

Two trained researchers independently screened the policy documents according to the predefined eligibility criteria. Any disagreements were resolved through discussion until consensus was reached. Ultimately, 52 policy documents were included in the analysis, including 7 national-level and 45 provincial-level documents. The retrieval and screening process is illustrated in Figure 1. Selected characteristics of the included policy documents are presented in Table 1, while the complete corpus is provided in Supplementary Table S1.

**Table 1:** Selected policy documents on fertility-friendly hospital development.

No.	Policy name	Issuing authority	Issue date	Administrative level	Document type
01	Notice on Issuing the Implementation Plan for the Healthcare Capacity Strengthening Project in the Inner Mongolia Autonomous Region	Health Commission of the Inner Mongolia Autonomous Region	2026-01-15	Provincial	Notice
02	Opinions on Promoting the Development of Fertility-Friendly Hospitals	National Health Commission and Three Other Departments	2024-12-24	National	Opinions
03	Notice on Strengthening the Management of Midwifery Services	National Health Commission	2024-03-16	National	Notice
04	Guiding Opinions on Further Improving and Implementing Measures to Support Proactive Childbearing	National Health Commission	2022-07-25	National	Opinions
05	Notice on Issuing the Action Plan for Enhancing Maternal and Infant Safety (2021–2025)	National Health Commission	2021-10-09	National	Notice
52	Notice on Strengthening Maternal Health Care under the Universal Two-Child Policy	Beijing Municipal Commission of Health and Family Planning	2016-04-07	Provincial	Notice

### 2.2 Development of the policy analysis framework

**Figure 2:**
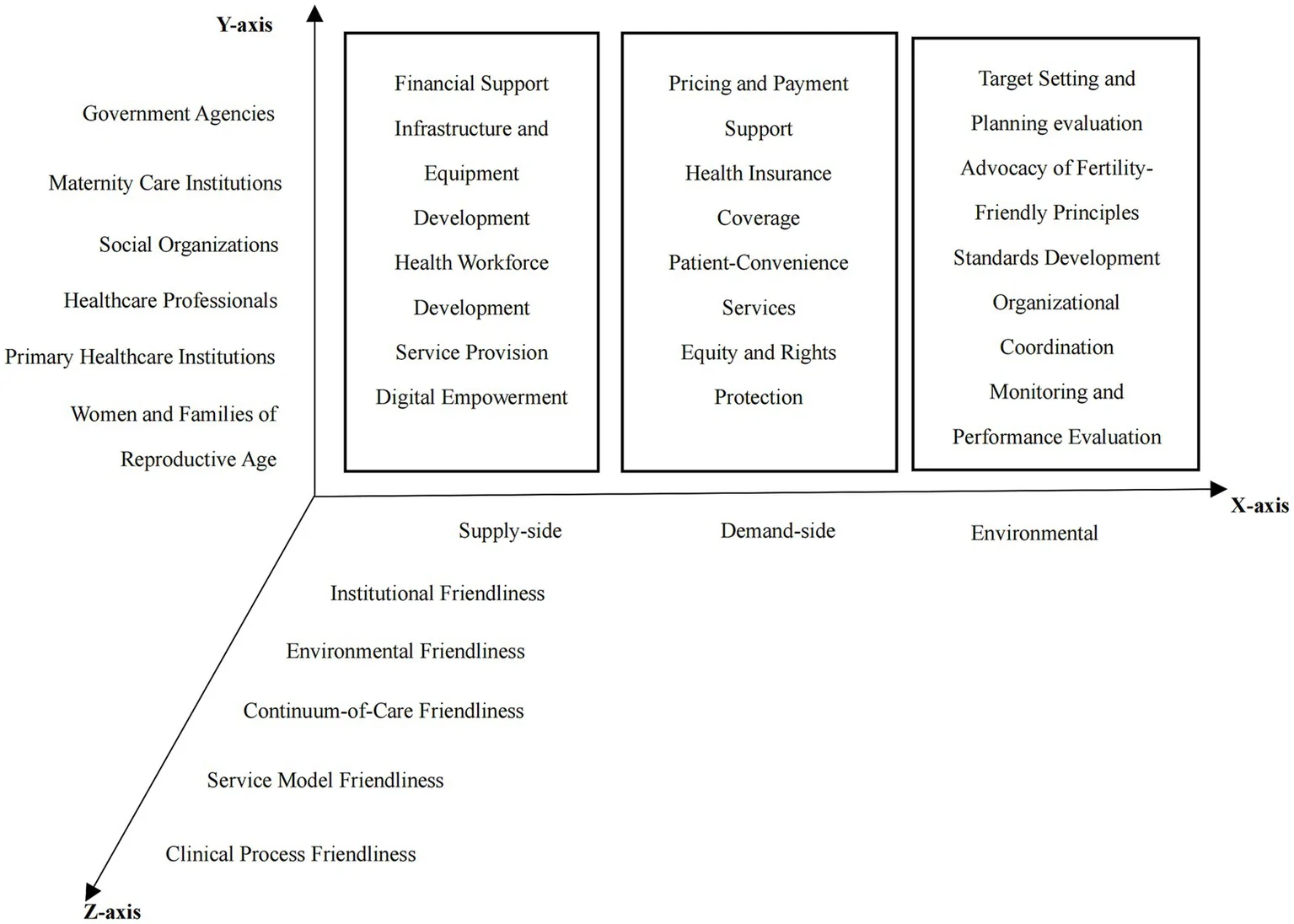
Three-dimensional analytical framework for fertility-friendly hospital development.

This study developed a three-dimensional analytical framework comprising policy tools, stakeholders, and policy objectives to characterize the structural features of fertility-friendly hospital policies from three perspectives: policy actions, participating actors, and intended policy outcomes. Policy tool analysis helps elucidate how governments use different policy instruments to facilitate stakeholder participation, coordinate diverse interests, and achieve policy objectives (10). Previous studies have also incorporated policy tools, stakeholders, and policy objectives into multidimensional policy text analysis frameworks (11, 12). Depending on research objectives, other studies have adopted alternative analytical dimensions, such as temporal dimensions for examining policy evolution or horizontal, vertical, and temporal dimensions for assessing policy synergy (13, 14). Given that this study focuses on the content structure of fertility-friendly hospital policies and the alignment among dimensions, these three dimensions were considered appropriate and sufficiently comprehensive to address the research questions and were therefore selected as the X-, Y-, and Z-dimensions, respectively (see Figure 2).

#### 2.2.1 X-dimension: policy tools

Policy tools connect policy objectives with their implementation (11). Drawing on the policy tool framework developed by Rothwell and Zegveld and considering the characteristics of policies on fertility-friendly hospital development, this study classified policy instruments into three categories: supply-side, demand-side, and environmental (14). Supply-side instruments directly support fertility-friendly hospital development by providing funding, improving facilities, and developing the health workforce. Demand-side instruments respond to the needs of service users through pricing support, health insurance coverage, public services, and measures to protect their rights and interests. Environmental instruments create supportive conditions for fertility-friendly hospital development by establishing standards and plans, strengthening supervision and evaluation, and promoting fertility-friendly principles. It should be noted that “environmental tools” in the policy-tool dimension refer to policy implementation mechanisms rather than the physical or service environments themselves. The definitions and specific components of each category are presented in Table 2.

**Table 2:** Classification of policy tools for fertility-friendly hospitals.

Category	Subcategory	Definition
Supply-side	Financial support	Provides financial support for fertility-friendly hospital development through government funding, earmarked funds, and construction subsidies
	Infrastructure and equipment development	Improves inpatient and childbirth conditions for pregnant and postpartum women by increasing obstetric bed capacity, renovating ward environments, and upgrading facilities and equipment
	Health workforce development	Enhances the capacity to provide fertility-related healthcare services by training and recruiting professionals in understaffed specialties, such as obstetrics and midwifery, and providing performance-based incentives
	Service provision	Provides women with a continuum of fertility-related healthcare services spanning preconception, antenatal care, childbirth, and postpartum care
	Digital empowerment	Improves the efficiency and patient experience of obstetric services through digital technologies, including online appointment systems, telemedicine, internet-based healthcare, and artificial intelligence
Demand-side	Pricing and payment support	Reduces out-of-pocket costs for fertility-related healthcare among disadvantaged groups through government subsidies, pricing adjustments, and financial assistance mechanisms
	Health insurance coverage	Reduces the financial burden of fertility-related healthcare by expanding health insurance coverage for eligible services
	Patient-convenience services	Improves access to healthcare by providing patient-centered services, such as advance scheduling for hospital admission and childbirth, one-stop administrative processing, and real-time settlement of medical expenses
	Equity and rights protection	Ensures that all population groups have equitable access to fertility-related healthcare and that their reproductive healthcare rights are protected
Environmental	Target setting and planning	Sets phased and measurable targets and specifies the implementation pathways and timelines for developing fertility-friendly hospitals across different levels of maternity care institutions
	Advocacy of Fertility-friendly principles	Promotes broader social recognition of fertility-friendly principles and fosters a supportive social environment
	Standards development	Provides technical guidance for fertility-friendly hospital development by establishing construction guidelines, service specifications, and clinical standards
	Organizational coordination	Establishes a coordinated maternal safety system through multidisciplinary collaboration, consultation, and referral mechanisms, and regional emergency care networks
	Monitoring and performance evaluation	Establishes a closed-loop management system through the development of evaluation criteria, satisfaction surveys, and tiered accreditation and review

#### 2.2.2 Y-dimension: stakeholders

Drawing on stakeholder theory (15) and previous policy analysis studies (12), the stakeholder dimension was used to identify the actors involved in policy formulation, implementation, service delivery, and policy receipt. Considering the functions and resource capacities of the actors involved in fertility-friendly hospital development, this study identified six key stakeholder groups. (1) Government agencies, including national and provincial health commissions, development and reform authorities, finance departments, and healthcare security authorities, are responsible for policy formulation, implementation, coordination, and oversight. (2) Maternity care institutions, including general hospitals, traditional Chinese medicine hospitals, maternal and child health hospitals, and obstetrics and gynecology hospitals, implement fertility-friendly hospital initiatives and deliver related services. (3) Healthcare professionals specializing in obstetrics, neonatology, nutrition, and related disciplines provide services spanning preconception assessment, pregnancy, childbirth, postpartum care, and neonatal care. (4) Women and families of reproductive age, including women of reproductive age, pregnant and postpartum women, and newborns, are the primary recipients and beneficiaries of these policy interventions. (5) Social organizations, including medical social workers and volunteers, provide companionship and practical support, extend health services, and facilitate cross-sector collaboration. (6) Primary healthcare institutions, including community health service centers and township health centers, support continuity of care and coordination at the community level.

#### 2.2.3 Z-dimension: policy objectives

Policy objectives refer to the intended goals and outcomes of policy formulation and implementation and indicate the directions toward which policy actions and instruments are oriented (11). The “Opinions on Promoting the Development of Fertility-Friendly Hospitals” require fertility-friendly principles to be fully incorporated into five areas: institutional arrangements, the physical environment, the continuum of care, service models, and clinical processes. Accordingly, this study classified the policy objectives for fertility-friendly hospital development into five dimensions: institutional friendliness, environmental friendliness, continuum-of-care friendliness, service model friendliness, and clinical process friendliness. In the policy-objective dimension, “environmental friendliness” refers to policy objectives aimed at creating supportive physical and service environments within healthcare institutions.

### 2.3 Text coding and analysis

The 52 included policy documents were coded and analyzed using NVivo 15 qualitative data analysis software. First, content directly related to fertility-friendly hospital development was extracted from each policy document and assigned a hierarchical code based on the document number, section, item, and text segment. For example, the code “04–2–1-3” refers to the third text segment under Item 1, “Improving the Continuum of Preconception, Maternal, and Child Health Services,” in section 2, “Improving the Quality of Preconception, Maternal, and Child Health Services,” of Policy Document 04, “Guiding Opinions on Further Improving and Implementing Measures to Support Proactive Childbearing.” The coded segment calls for strengthening the capacity of maternal and child health institutions and establishing government-run standardized institutions at the provincial, municipal, and county levels. Second, three sets of nodes were created in accordance with the analytical framework: policy tools, stakeholders, and policy objectives. In the example above, the text segment was coded as a supply-side tool under “infrastructure and equipment development,” with “maternity care institutions” identified as the stakeholder group and “environmental friendliness” as the policy objective. Third, coding followed the principle of the smallest meaningful unit. A text segment expressing one coherent meaning was treated as a single coding unit. When a segment contained multiple meanings, it was progressively divided until no further meaningful subdivision was possible (16). A single text excerpt could be coded across multiple analytical dimensions according to its policy content; when multiple independent categories were identified within the same dimension, multiple codes were assigned (10). Consequently, the total for each dimension represents the number of coded references rather than the number of unique text excerpts. Percentages within each dimension were calculated using the corresponding number of coded references as the denominator and were interpreted only within each dimension rather than directly compared across dimensions. The complete coding framework, including operational definitions, decision rules, and representative coding examples, is provided in Supplementary Table S2.

### 2.4 Inter-coder reliability assessment

After independent coding was completed, the “Coding Comparison Query” function in NVivo 15 was used to compare the coding results between the two researchers. To ensure the representativeness of the reliability assessment sample, a stratified random sampling method was used to select 20 policy documents (38.5%) from the 52 included documents for double coding, comprising 5 national-level and 15 provincial-level policy documents. The remaining 32 policy documents were coded independently by the first researcher. The reliability assessment included 1,002 coding references from the 20 double-coded documents, including 366 references for the policy tools dimension, 325 references for the stakeholder dimension, and 311 references for the policy objectives dimension. The NVivo Coding Comparison Query results showed a Cohen’s κ coefficient of 0.866 and an overall percentage agreement of 99.89%, indicating excellent agreement between the two coders. Detailed overall and dimension-specific reliability results are presented in Supplementary Table S3.

Coding discrepancies primarily arose from policy provisions with overlapping functional boundaries across coding categories. Some policy provisions simultaneously involved multiple policy functions or stakeholder groups, resulting in differences in category assignment between coders. To resolve these discrepancies, the two researchers reviewed the relevant text excerpts according to the predefined coding framework and decision rules. Final coding decisions were made by considering the primary policy functions and core governance intentions reflected in the texts. When consensus could not be reached, a third researcher made the final decision according to the established coding rules.

## 3 Results

### 3.1 Characteristics of included policy documents

#### 3.1.1 Distribution by policy period

In December 2015, the Population and Family Planning Law of the People’s Republic of China was amended, and the universal two-child policy came into effect on 1 January 2016 (17). In 2021, China’s fertility policy underwent another major adjustment. The “Decision of the Central Committee of the Communist Party of China and the State Council on Optimizing the Fertility Policy to Promote Long-term Balanced Population Development” explicitly introduced the three-child policy and associated supporting measures (6). The Population and Family Planning Law of the People’s Republic of China, amended in the same year, further established the three-child policy in law and introduced measures to reduce the financial burden of childbirth, child-rearing, and education on families (17). Based on this policy evolution, 2021 was used as a key milestone marking the transition from the universal two-child policy to the three-child policy. The 52 included policy documents were accordingly divided into two periods: the universal two-child policy period (2016–2020) and the three-child policy and fertility-supportive development period (2021–February 2026).

Three policy documents (5.77%) were included during 2016–2020, all of which were provincial-level policies. During 2021–February 2026, 49 documents (94.23%) were included, comprising seven national-level and 42 provincial-level policies. The included policies were therefore concentrated in the period from 2021 onward, and all national-level policies were issued during this period. Given the small number of documents included during 2016–2020 (*n* = 3), no further comparison between the two periods was conducted for the distributions of policy tools, stakeholders, or policy objectives. Accordingly, the overall findings should be interpreted as an aggregate distributional pattern within the included policy corpus rather than as persistent structural characteristics of the policy system throughout 2016–2026.

#### 3.1.2 Distribution by administrative level and provincial coverage

The 45 provincial-level documents represented 23 provincial-level administrative regions, with 7 from Beijing, 5 from Inner Mongolia, 4 each from Guizhou and Jiangsu, 2 each from Fujian, Guangxi, Jiangxi, Ningxia, Shaanxi, and Yunnan, and 1 each from the remaining 13 regions. Under the predefined search strategy and eligibility criteria, no eligible provincial-level policy documents were identified for eight provincial-level administrative regions: Anhui, Gansu, Guangdong, Liaoning, Sichuan, Tianjin, Tibet, and Xinjiang. The distribution of included policy documents by policy period and administrative level is presented in Table 3.

**Table 3:** Distribution of included policy documents by policy period and administrative level.

Policy period	National-level, n	Provincial-level, n	Total, n (%)
Universal two-child policy period (2016–2020)	0	3	3 (5.77)
Three-child policy and fertility-support development period (2021–February 2026)	7	42	49 (94.23)
Total	7	45	52 (100.00)

Percentages were calculated using the total number of included policy documents (*n* = 52) as the denominator.

### 3.2 Distribution of policy tools

The coding analysis yielded 516 coded references (see Table 4). All three categories of policy tools—supply-side, environmental, and demand-side—were represented in the policy documents, although their distribution was markedly uneven. Supply-side tools accounted for the largest proportion of coded references (53.29%), followed by environmental tools (34.69%), whereas demand-side tools accounted for only 12.02%. A document-level sensitivity analysis showed that supply-side, environmental, and demand-side policy tools were identified in 49 (94.23%), 40 (76.92%), and 36 (69.23%) of the 52 policy documents, respectively. This ordering was consistent with the coded-reference frequency analysis. These findings indicate that the included policy texts placed greater emphasis on supply-side and environmental tools. These tools primarily focus on expanding fertility-related healthcare capacity, strengthening obstetric infrastructure and workforce capacity, promoting multidisciplinary collaboration, and improving maternal and infant safety systems. In contrast, demand-side tools were comparatively less frequently represented in the pooled policy corpus.

**Table 4:** Frequency distribution of policy tools (*n* = 516).

Category	Subcategory	Number of coded references, n	Percentage (%)
Supply-side (53.29%)	Financial support	7	1.36
	Infrastructure and equipment development	60	11.63
	Health workforce development	41	7.95
	Service provision	142	27.52
	Digital empowerment	25	4.84
Demand-side (12.02%)	Pricing and payment support	23	4.46
	Health insurance coverage	19	3.68
	Patient-convenience services	11	2.13
	Equity and rights protection	9	1.74
Environmental (34.69%)	Target setting and planning	21	4.07
	Advocacy of fertility-friendly principles	10	1.94
	Standards development	21	4.07
	Organizational coordination	93	18.02
	Monitoring and performance evaluation	34	6.59
Total		516	100.00

Percentages were calculated using the total number of policy-tool coded references (*n* = 516) as the denominator. All percentages were rounded to two decimal places.

Supply-side policy tools were the most frequently represented category, with greater emphasis on service provision. Among the supply-side subcategories, service provision accounted for the largest proportion (27.52%), followed by infrastructure and equipment development (11.63%) and health workforce development (7.95%). Digital empowerment (4.84%) and financial support (1.36%) accounted for smaller shares, indicating comparatively less textual emphasis on these areas.

Environmental policy tools primarily emphasized organizational coordination. Environmental tools accounted for 34.69% of policy-tool coded references, ranking second among the three categories of policy tools. Among the environmental tool subcategories, “organizational coordination” accounted for the largest proportion of coded references (18.02%), followed by “monitoring and performance evaluation” (6.59%). Standards development and target setting and planning each accounted for 4.07%, whereas “advocacy of fertility-friendly principles” represented the smallest proportion (1.94%). These findings suggest that current policies emphasize organizational coordination through measures such as implementing the Five Strategies for Maternal and Newborn Safety (FSMNS), a national framework established in China to strengthen maternal and newborn safety management through coordinated healthcare services (18); establishing regional care networks for critically ill pregnant and postpartum women; and developing multidisciplinary care mechanisms. Although policy documents generally advocated fertility-friendly principles, few specified structured communication strategies or mechanisms for evaluating their effectiveness, indicating that advocacy-oriented measures received comparatively limited attention in the policy corpus.

Demand-side policy tools were less frequently represented. Demand-side tools accounted for only 12.02% of policy-tool coded references, the smallest share among the three policy tool categories. Among the demand-side subcategories, “pricing and payment support” accounted for the largest share of all coded references (4.46%), followed by “health insurance coverage” (3.68%), “patient-convenience services” (2.13%), and “equity and rights protection” (1.74%). Demand-side tools mainly seek to improve access to fertility-related healthcare by reducing out-of-pocket costs and expanding health insurance coverage. However, comparatively limited attention was given to patient-convenience services and the protection of reproductive healthcare rights.

### 3.3 Representation of stakeholders in policy documents

**Figure 3:**
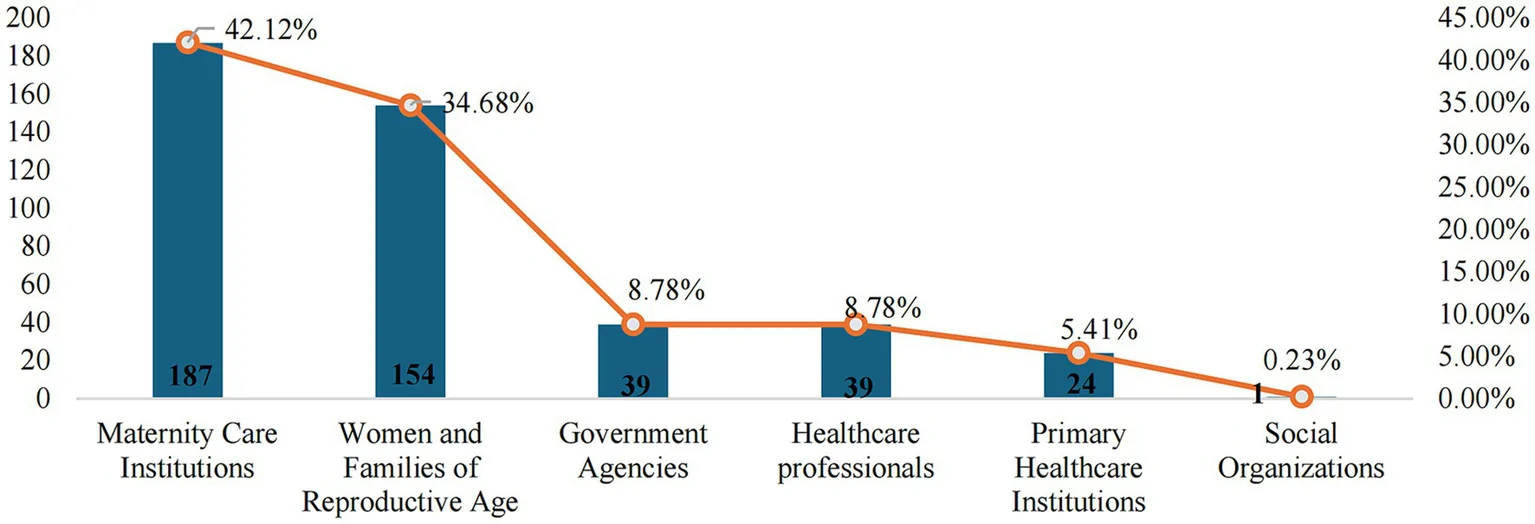
Distribution of stakeholders represented in policy documents.

The stakeholder analysis yielded 444 coded references. Maternity care institutions accounted for the largest share of stakeholder-related coded references (42.12%), followed by women and families of reproductive age (34.68%). This distribution suggests that the policies primarily focused on the provision of fertility-related healthcare through maternity care institutions, with women and families of reproductive age as the principal beneficiaries. Government agencies and healthcare professionals each accounted for 8.78%, while primary healthcare institutions and social organizations represented 5.41 and 0.23%, respectively (see Figure 3). Overall, the stakeholder structure was centered on maternity care institutions and women and families of reproductive age, with comparatively limited representation of government agencies, healthcare professionals, and primary healthcare institutions. Social organizations were rarely represented, indicating comparatively limited explicit policy attention to broader social participation and cross-sector collaboration.

### 3.4 Distribution of policy objectives

**Figure 4:**
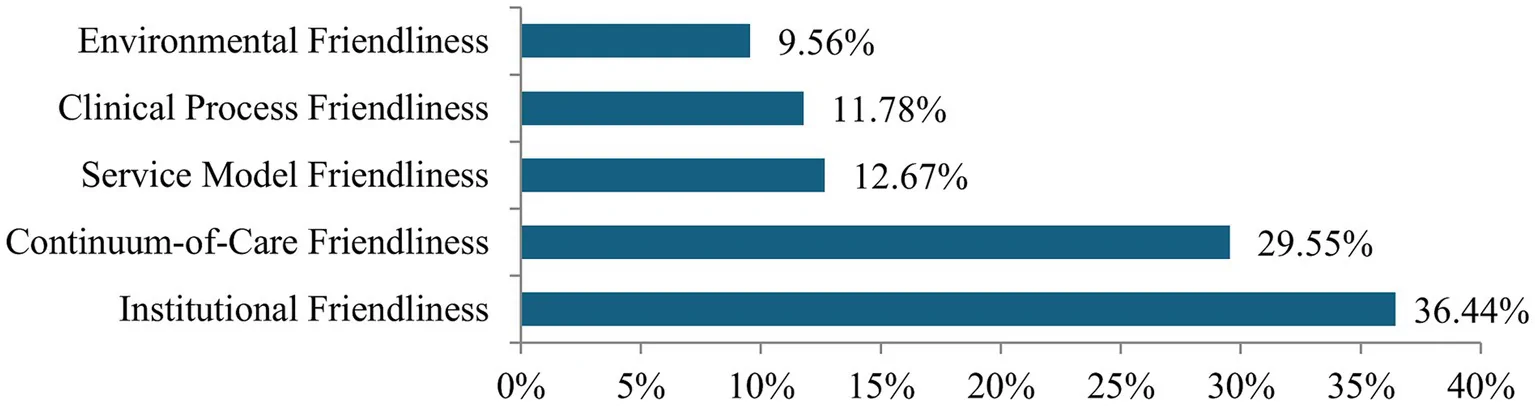
Distribution of policy objectives.

The analysis of the policy-objective dimension (Z-dimension) identified 450 coded references, showing an uneven distribution across the five objectives (Figure 4). Institutional friendliness accounted for the largest proportion, with 164 coding references (36.44%). These policies mainly involved implementing the Five Strategies for Maternal and Newborn Safety (FSMNS), establishing critical maternal care networks, and developing multidisciplinary collaborative care mechanisms. Continuum-of-care friendliness ranked second, with 133 coding references (29.55%). These policies focused on improving fertility-related healthcare services, including antenatal examinations, hospital delivery, and postpartum care, which were related to women’s childbirth experiences. Service model friendliness and clinical process friendliness accounted for 57 (12.67%) and 53 (11.78%) coding references, respectively. Service model friendliness mainly covered nutritional guidance and mental health services during pregnancy and the postpartum period. Clinical process friendliness included improving clinical management systems, advancing the digitalization of obstetric services, and providing remote consultations and online referrals through the Maternal and Child Health Telecare Cloud Platform. Environmental friendliness accounted for the smallest proportion, with 43 coding references (9.56%), and mainly involved improving maternity ward environments and establishing one-stop service centers. Overall, coding references were concentrated on institutional friendliness and continuum-of-care friendliness, which together accounted for the majority of all coding references.

### 3.5 Cross-dimensional alignment

#### 3.5.1 Policy tools and stakeholders

Maternity care institutions were most frequently represented in the cross-dimensional coding, while women and families of reproductive age were the principal beneficiary group. The three categories of policy tools were associated with most stakeholder groups. Environmental tools were most frequently associated with government agencies, maternity care institutions, and primary healthcare institutions, whereas supply-side tools were primarily associated with women and families of reproductive age and healthcare professionals. In contrast, social organizations were not represented in the cross-dimensional coding of policy tools and stakeholders. The cross-dimensional distribution is shown in Supplementary Figure S1.

#### 3.5.2 Policy tools and policy objectives

The cross-analysis of policy tools and policy objectives showed that policy tool coding units were distributed markedly unevenly across the five objectives of fertility-friendly hospital development. Institutional friendliness and continuum-of-care friendliness were associated with the largest numbers of policy tool coding units. Environmental tools predominated within institutional friendliness, whereas supply-side tools were most frequently associated with continuum-of-care friendliness. Clinical process friendliness and service model friendliness were associated with fewer policy tool coding units, which were predominantly demand-side and supply-side tools, respectively. Environmental friendliness had the fewest policy tool coding units, most of which were supply-side tools. The results of the cross-analysis are presented in Supplementary Figure S2.

#### 3.5.3 Stakeholders and policy objectives

Maternity care institutions accounted for the largest share under institutional friendliness (103/178, 57.88%), whereas women and families of reproductive age accounted for the largest share under continuum-of-care friendliness (110/144, 76.39%). The distribution of stakeholder groups across the five policy objectives is presented in Table 5. The findings suggest that current policies position maternity care institutions as the principal actors associated with institutional friendliness and environmental friendliness. In contrast, women and families of reproductive age were the primary beneficiary group associated with continuum-of-care friendliness and clinical process friendliness.

**Table 5:** Results of the Y–Z cross-analysis.

Policy objectives	Primary healthcare institutions		Social organizations		Healthcare professionals		Women and families of reproductive age		Government agencies		Maternity care institutions	
	Frequency, n	Percentage (%)	Frequency, n	Percentage (%)	Frequency, n	Percentage (%)	Frequency, n	Percentage (%)	Frequency, n	Percentage (%)	Frequency, n	Percentage (%)
Service model friendliness (12.60%)	2	3.28	1	1.64	19	31.15	16	26.23	0	0	23	37.70
Environmental friendliness (9.30%)	0	0	0	0	1	2.22	7	15.56	3	6.67	34	75.56
Institutional friendliness (36.78%)	4	2.25	0	0	22	12.36	14	*7.87*	35	19.66	103	57.87
Continuum-of-Care friendliness (29.75%)	3	2.08	0	0	0	0	110	76.39	*2*	1.39	29	20.14
Clinical process friendliness (11.57%)	15	26.79	0	0	0	0	21	37.50	*0*	0	20	35.71

Percentages were calculated using the total number of coded references within each policy objective as the denominator. All percentages were rounded to two decimal places.

#### 3.5.4 Policy tools, stakeholders, and policy objectives

**Figure 5:**
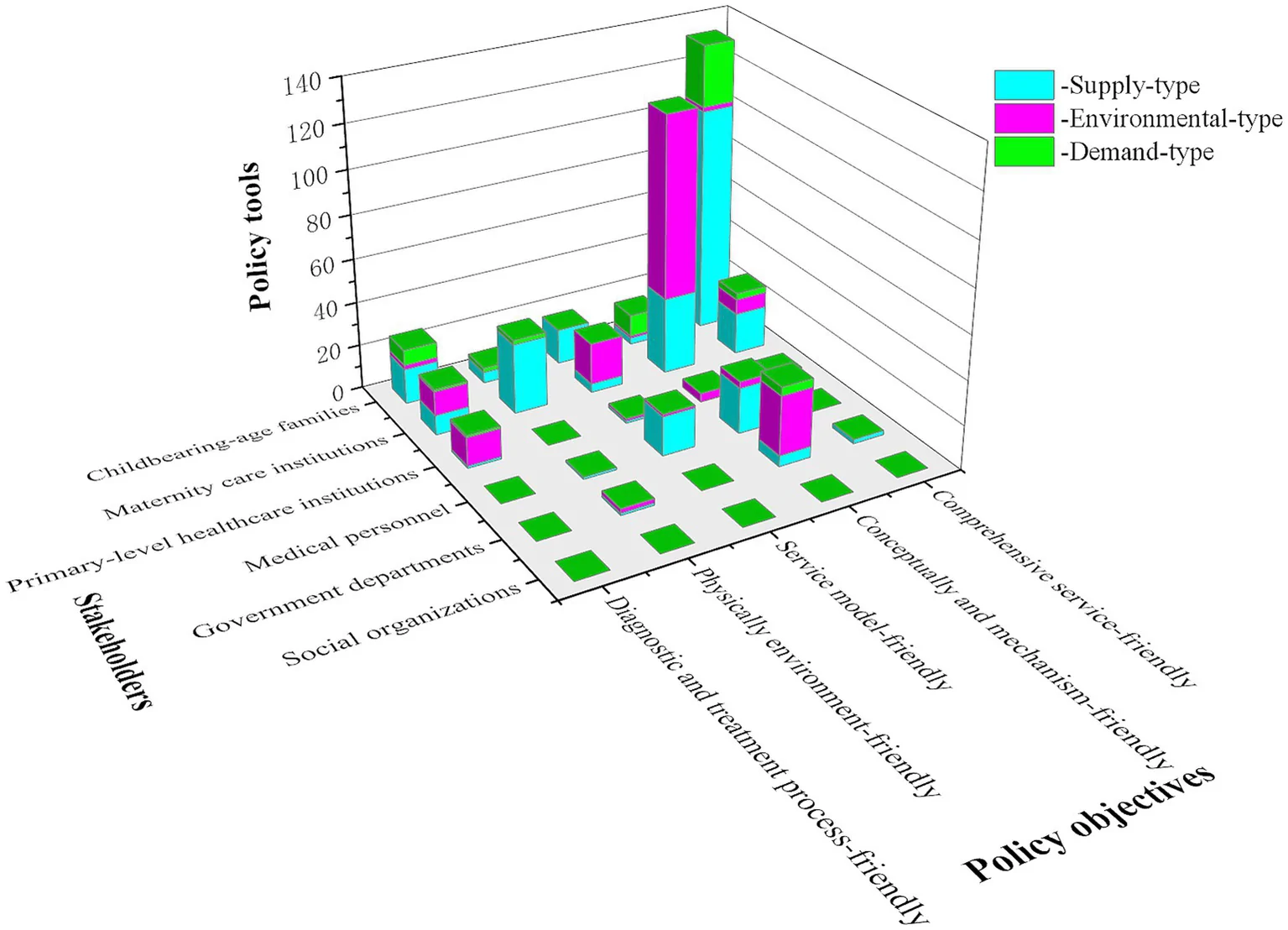
Three-dimensional cross-analysis of policy tools, stakeholders, and policy objectives.

A three-dimensional cross-analysis was conducted to explore the relationships among policy tools, stakeholders, and policy objectives. Within the supply-side tool category, women and families of reproductive age accounted for the largest proportion under the continuum-of-care friendliness objective (103/126, 81.7%). This finding suggests that supply-side tools primarily addressed the needs of women and families of reproductive age by improving fertility-related healthcare across the continuum of care. Maternity care institutions also showed a substantial proportion under the institutional friendliness objective (35/64, 54.7%). Within the environmental tool category, maternity care institutions accounted for the largest proportion under the institutional friendliness objective (84/119, 70.6%). This pattern highlights the prominent role of environmental tools in promoting institutional coordination, monitoring, and performance evaluation. Within the demand-side tool category, women and families of reproductive age accounted for the highest proportion under the continuum-of-care friendliness objective (28/31, 90.3%). These tools mainly included health insurance coverage and pricing and payment support, aiming to reduce the financial burden associated with fertility-related healthcare. The results of the three-dimensional cross-analysis are presented in Figure 5.

### 3.6 Comparison between national- and provincial-level policy documents

Regarding policy tools, national- and provincial-level policy documents (Table 6) showed similar distribution patterns, with supply-side policy tools being the predominant category, followed by environmental policy tools and demand-side policy tools. In provincial-level policy documents, supply-side policy tools accounted for the largest proportion (53.50%), followed by environmental policy tools (34.58%) and demand-side policy tools (11.92%). In national-level policy documents, the corresponding proportions were 52.27, 35.23, and 12.50%, respectively. At the subcategory level, differences were observed in service provision and organizational coordination. Specifically, service provision and organizational coordination accounted for 28.27 and 19.39%, respectively, in provincial-level policy documents, compared with 23.86 and 11.36%, respectively, in national-level policy documents (Supplementary Table S4).

**Table 6:** Comparison of three policy dimensions between national- and provincial-level policy documents.

Dimension	Category	Provincial-level, n (%)	National-level n, (%)
X-dimension: policy tools	Supply-side	229 (53.50%)	46 (52.27%)
	Environmental	148 (34.58%)	31 (35.23%)
	Demand-side	51 (11.92%)	11 (12.50%)
Y-dimension: stakeholders	Maternity care institutions	156 (42.16%)	31 (41.89%)
	Women and families of reproductive age	129 (34.86%)	25 (33.78%)
	Government agencies	28 (7.57%)	11 (14.86%)
	Healthcare professionals	33 (8.92%)	6 (8.11%)
	Primary healthcare institutions	23 (6.22%)	1 (1.35%)
	Social organizations	1 (0.27%)	0 (0.00%)
Z-dimension: policy objectives	Institutional friendliness	142 (37.47%)	22 (30.99%)
	Continuum-of-care friendliness	112 (29.55%)	21 (29.58%)
	Environmental friendliness	34 (8.97%)	9 (12.68%)
	Service model friendliness	49 (12.93%)	8 (11.27%)
	Clinical process friendliness	42 (11.08%)	11 (15.49%)

Percentages were calculated using the total number of coded units within each dimension as the denominator. The denominators were 428 and 88 coded units for policy tools, 370 and 74 coded units for stakeholders, and 379 and 71 coded units for policy objectives in provincial- and national-level documents, respectively.

Regarding stakeholders, both national- and provincial-level policy documents primarily focused on maternity care institutions and women and families of reproductive age. In addition, government departments accounted for a higher proportion in national-level policy documents (14.86%) than in provincial-level policy documents (7.57%).

Regarding policy objectives, both levels of policy documents primarily focused on institutional friendliness and continuum-of-care friendliness. Further comparison showed that clinical process friendliness and environmental friendliness accounted for higher proportions in national-level policy documents (15.49 and 12.68%, respectively) than in provincial-level policy documents (11.08 and 8.97%, respectively). In contrast, institutional friendliness accounted for a higher proportion in provincial-level policy documents (37.47%) than in national-level policy documents (30.99%).

## 4 Discussion

### 4.1 Uneven distribution of the policy tool mix

#### 4.1.1 Policy emphasis within supply-side tools: greater attention to service provision than to digital and financial support

Supply-side tools constituted the largest component of the policy tool mix. Among the supply-side subcategories, service provision accounted for the largest share of policy-tool coded references (27.52%), consistent with the central objective of fertility-friendly hospital development: improving women’s childbirth experiences. As fertility-friendly principles have received increasing policy attention, medical institutions have continued to improve obstetric service environments, standardize the provision of labor analgesia, and enhance women’s care experiences (19). This trend is consistent with the predominance of supply-side tools in China’s policies on fertility-friendly hospital development. Digital technologies have increasingly been applied in healthcare (20), with emerging applications in maternal and child health services, including maternal health management and neonatal health monitoring (20–24). These applications may provide additional support for improving the delivery of maternal and child healthcare services. However, digital empowerment accounted for only 4.84% of all coding references, indicating comparatively less textual emphasis on digital-related policy measures. The policy documents also contained relatively limited detail on standards for the development of obstetric information systems; however, the present content analysis cannot determine whether this level of policy specification is adequate for implementation. Although current policies support the use of the Maternal and Child Health Telecare Cloud Platform, a national telemedicine platform that facilitates remote consultation, training, clinical guidance, and bidirectional referral among maternal and child healthcare institutions (23), the development and maintenance of such digital systems require substantial investment to enhance the coordination and continuity of maternal and child healthcare services. However, because this study assessed policy-text references rather than actual expenditure, the relatively low frequency of financial-support coding should not be interpreted as evidence of insufficient investment. Future implementation studies are needed to determine whether existing funding arrangements, technical standards, and digital infrastructure are sufficient to support the effective implementation of digital maternal and child healthcare services. Based on such evidence, future policy deliberation could consider further strengthening digital-related measures in fertility-friendly hospital development.

#### 4.1.2 Environmental tools emphasized organizational coordination more than advocacy of fertility-friendly principles

Among environmental policy tools, organizational coordination was the dominant subcategory, whereas advocacy of fertility-friendly principles accounted for only 1.94% of all coding units. This pattern suggests that current policies place greater emphasis on defining the institutional responsibilities and service requirements of maternity care institutions than on communication-oriented and flexible policy guidance. Although these institutional arrangements may help safeguard service quality for pregnant and postpartum women, advocacy-oriented measures were less frequently represented in the policy corpus. In addition, some local hospital quality evaluation systems have incorporated indicators related to fertility-friendly hospital development (25). Based on these findings, future policies could further strengthen advocacy-oriented measures by improving information dissemination mechanisms, enhancing public understanding of fertility-friendly hospital services, and appropriately leveraging digital communication channels to promote awareness of the fertility-friendly concept. Such measures may help improve public awareness and foster a more supportive social environment for fertility-friendly hospital development.

#### 4.1.3 Demand-side policy instruments were relatively infrequent in policy texts, accounting for 12.02% of policy instrument coding references

Policies primarily focused on price and payment support, as well as health insurance coverage. These instruments primarily included measures aimed at reducing out-of-pocket expenses for fertility-related healthcare services and may help improve access to reproductive and maternal healthcare services for pregnant and postpartum women. The “Opinions on Promoting the Development of Fertility-Friendly Hospitals” propose including eligible labor analgesia services and assisted reproductive technology programs within the scope of medical insurance reimbursement, highlighting the potential role of financial protection measures in reducing barriers to fertility-related healthcare services (3). In light of previous research (26), future policy research could further explore how to optimize the allocation and coordination of demand-side tools across different stages of care to support more continuous and comprehensive coverage of reproductive and maternal healthcare services. Furthermore, the findings indicate that demand-side policy instruments placed relatively limited emphasis on equity and rights protection. Equitable maternity protection is important for safeguarding women’s reproductive rights and supporting fertility intentions (27). Previous studies have shown that low-income women may face multiple structural barriers to accessing antenatal and postpartum care services, including inadequate health insurance coverage and barriers to healthcare access (28). International evidence further suggests that maternal and perinatal risks are multidimensional and may vary across population groups, highlighting the importance of considering population heterogeneity when developing policies to promote equity in pregnancy and childbirth (29). Future policy formulation and implementation research could explore whether expanding coverage of fertility-related healthcare services and fertility support programs may improve service accessibility, equity, and financial protection. Future studies could also examine whether strengthening policy navigation support and assistance mechanisms may improve pregnant women’s awareness of available support policies and facilitate access to relevant services. Further refinement of the fertility support system and family support mechanisms may represent an important direction for protecting women’s reproductive rights and optimizing the fertility support environment (30).

### 4.2 Advancing multi-stakeholder collaboration and collaborative governance

As an important component of the fertility support policy system, fertility-friendly hospitals are expected to achieve multiple policy objectives. Their development, therefore, requires coordinated participation across government departments, healthcare institutions, professional groups, and social organizations. However, the stakeholder analysis showed that maternity care institutions accounted for 42.12% of stakeholder-related coded references, followed by women and families of reproductive age (34.68%). Healthcare professionals (8.78%), primary healthcare institutions (5.41%), and social organizations (0.23%) were less frequently represented in the policy corpus. This finding indicates that existing policy documents give comparatively limited attention to healthcare professionals, primary healthcare institutions, and social organizations. However, the low frequency of references to a particular stakeholder group should not be interpreted as direct evidence of inadequate incentives, service capacity, or participation in practice. Given the important roles of healthcare professionals, primary healthcare institutions, and social organizations in professional service delivery, community-based continuity of care, and supportive services such as psychological support, nutritional support, and postpartum care, their comparatively limited representation in the policy corpus warrants greater attention in future policy development.

Accordingly, future policy development may consider further clarifying the roles of and support mechanisms for healthcare professionals, primary healthcare institutions, and social organizations in fertility-friendly hospital development, while promoting broader multi-stakeholder participation and establishing clearer coordination mechanisms. Implementation-focused research is also needed to assess these stakeholders’ actual participation, resource allocation, and needs during policy implementation, thereby providing an empirical basis for developing a more coordinated multi-stakeholder support framework.

### 4.3 Distribution of policy tools across policy objectives

Previous research has shown that the allocation of policy tools is often uneven across policy objectives (31). In the present study, institutional friendliness relied predominantly on environmental tools, whereas continuum-of-care friendliness relied mainly on supply-side tools. By contrast, service model friendliness, clinical process friendliness, and environmental friendliness were associated with fewer policy-tool coded references across the three policy-tool categories. This distribution indicates that policy attention in the included documents was concentrated on institutional development and continuum-of-care objectives, while the other objectives were associated with comparatively fewer policy-tool coded references. This pattern may reflect the emphasis placed on institutional development and system improvement during the policy development period examined (32). However, because the included documents span different stages of China’s fertility-policy development, the observed distribution should be interpreted as an aggregate pattern within the 2016–2026 policy corpus rather than as a persistent structural characteristic of the policy system. Future policies may benefit from a more diversified combination of policy tools, including greater integration of supply-side support, demand-side incentives, and environmental measures across service model friendliness, clinical process friendliness, and environmental friendliness.

### 4.4 Administrative-level differences in fertility-friendly hospital policies

Within the three-dimensional analytical framework, the national and provincial policy documents included in this study showed similar distribution patterns across major policy instruments, stakeholders, and policy objectives, although differences remained in selected categories. However, comparisons between national and provincial policies primarily reflect differences between governance levels and do not fully capture regional heterogeneity within individual provinces. Existing research indicates that provincial fertility support policies vary across regions of China in terms of policy instrument selection and allocation (33). Chinese provinces differ in economic development, demographic structure, fiscal capacity, and healthcare resources; these contextual factors may be associated with variations in local policy priorities, policy instrument selection, and objective setting. For example, differences in fiscal capacity may influence local governments’ policy implementation capacity and available policy space (34), while variations in population mobility may alter local demand for public services and resource allocation priorities (35). Consequently, the distribution of provincial policy instruments observed in this study may be influenced by regional contextual factors and should not be interpreted as fully representative of policy mixes across all provinces.

Among the policy documents included in this study, both administrative levels predominantly employed supply-side policy instruments, while demand-side policy instruments accounted for a relatively small proportion, indicating similar patterns in policy instrument distribution across the two levels. However, differences between governance levels in policy instrument subcategories and stakeholder representation may reflect the distinct functions of central and provincial departments in policy formulation, coordination, and implementation (36). Consequently, the findings of this study are primarily applicable to comparisons of policy characteristics across governance levels and should not be directly generalized to specific provincial policy models. Future research could incorporate regional socioeconomic, demographic, fiscal, and healthcare resource indicators to conduct stratified analyses and further explore variations in policy instrument allocation for fertility-friendly hospital development across different regional contexts.

### 4.5 Patterns of cross-dimensional alignment

The three-dimensional cross-analysis showed that, under key objectives such as institutional friendliness and environmental friendliness, substantially more policy tool coding units were associated with maternity care institutions than with other stakeholder groups. This disparity reflects an uneven pattern of cross-dimensional association among policy tools, stakeholders, and policy objectives. Healthcare professionals and primary healthcare institutions were less frequently represented in the three-dimensional coding combinations. Healthcare professionals appeared only in coding combinations involving health workforce development and service model friendliness, whereas primary healthcare institutions were represented only under clinical process friendliness. This narrow distribution indicates that cross-dimensional associations were concentrated within a relatively small number of stakeholder–tool–objective combinations. Social organizations were infrequently represented, despite their potential to contribute to psychological support, health education, and patient accompaniment for pregnant and postpartum women (37). These patterns have implications for future policy development, particularly for stakeholder participation and the integration of policy tools. First, future policies may consider increasing explicit attention to healthcare professionals, primary healthcare institutions, and social organizations to promote broader multi-stakeholder participation and collaborative governance (11). Second, a broader and more integrated use of supply-side and demand-side tools may be considered across service model friendliness, clinical process friendliness, and environmental friendliness (26). These approaches may contribute to more diversified cross-dimensional alignment among policy tools, stakeholders, and policy objectives.

This study has several limitations. First, policy documents primarily reflect formal policy intentions and cannot directly indicate the extent or effectiveness of policy implementation. Accordingly, coding frequencies should be interpreted as indicators of relative textual emphasis within the policy corpus rather than as direct measures of policy adequacy, actual expenditure, coordination quality, or implementation effectiveness. Second, given the cross-sectional design of this policy text analysis, the study could not capture temporal changes or dynamic evolution in policy characteristics. Future studies should employ longitudinal or period-stratified analyses to further examine changes in policy tools, stakeholders, and policy objectives across different policy stages. Finally, national-and provincial-level policies may differ in their priorities and implementation pathways, while provincial variation in economic development, population structure, and fiscal capacity may influence policy content and implementation conditions. Future studies should incorporate longitudinal policy analysis, comparisons between national- and provincial-level policies, cross-provincial comparisons, and implementation evaluations.

## 5 Conclusion

This study employed a three-dimensional analytical framework to analyze 52 national- and provincial-level policy documents related to the development of fertility-friendly hospitals in China. Policy text coding showed that supply-side and environmental tools predominated, whereas demand-side tools were less frequently represented. Policy attention was concentrated on maternity care institutions and women and families of reproductive age, while healthcare professionals, primary healthcare institutions, and social organizations received comparatively less textual representation. Policy objectives primarily focused on institutional frameworks and continuum-of-care support. These findings describe patterns of policy emphasis within the analyzed documents rather than providing direct evidence of policy adequacy or implementation effectiveness. Future policy refinement may further explore whether broader integration of demand-side tools, digital-related measures, and stakeholder participation could enhance the comprehensiveness of fertility-friendly hospital policies. Future research should conduct implementation evaluations and regional comparative analyses to further examine whether and how these policies influence the accessibility, equity, and quality of reproductive and maternal healthcare services.

## Data Availability

The original contributions presented in the study are included in the article/Supplementary material, further inquiries can be directed to the corresponding author.

## Supplementary material

Supplementary Figure S1 Results of the X–Y cross-analysis.

Supplementary Figure S2 Results of the X–Z cross-analysis.

Supplementary Figure S3 Word cloud of thematic terms.
