# Supplementary material for: A quantitative content analysis of Chinese policies on fertility-friendly hospital development: a three-dimensional framework
Source: Front Public Health. 2026 Sep 4;14:1953541. doi: 10.3389/fpubh.2026.1953541 (PMC13585940; doi:10.3389/fpubh.2026.1953541)
Supplement: Supplementary file 1 [file Data_sheet_1.zip › Supplementary Material/Supplementary File S1. Exploratory word-frequency analysis.docx]

**Supplementary File S1. Exploratory word-frequency analysis**

**Methods**

As an exploratory supplementary analysis, word-frequency analysis was conducted on the original Chinese texts of the included policy documents using the Word Frequency Query in NVivo 15. The project text-content language was set to Chinese (China). The query was configured to identify the 50 most frequent terms, with the minimum word length set at two characters. The default Chinese stop-word list in NVivo 15 was retained without manual modification. No external Chinese word-segmentation software or dictionary was used, and no manual synonym merging was performed. Terms were retained as generated by the NVivo Word Frequency Query. The query output included term length, frequency count, and weighted percentage. For concise presentation, Supplementary Table S5 presents the 20 highest-frequency terms among the 50 terms identified by the query, together with the original Chinese terms, English translations, frequencies, and weighted percentages. The corresponding word cloud is presented in Supplementary Figure S3 and is used solely as an exploratory visualization.
**Results**

The most frequently occurring terms were “services” (n = 1,311), “pregnant and postpartum women” (n = 939), “institutions” (n = 925), “health care” (n = 830), and “medical care” (n = 821). “Obstetrics” and “childbirth” each occurred 454 times. These frequencies were used solely to provide a descriptive overview of the policy corpus and were not interpreted as indicators of policy adequacy, implementation intensity, or unmet policy needs.

Supplementary Table S5 NVivo word-frequency analysis

| Thematic Term | English translation | Frequency | Weighted percentage (%) | Thematic Term | English translation | Frequency | Weighted percentage (%) |
| --- | --- | --- | --- | --- | --- | --- | --- |
| 服务 | Services | 1311 | 2.55 | 分娩 | Childbirth | 454 | 0.88 |
| 孕产妇 | Pregnant and Postpartum Women | 939 | 1.83 | 救治 | Treatment | 432 | 0.84 |
| 机构 | Institutions | 925 | 1.80 | 助产 | Midwifery | 400 | 0.78 |
| 保健 | Health Care | 830 | 1.61 | 医院 | Hospital | 373 | 0.73 |
| 医疗 | Medical Care | 821 | 1.60 | 危重 | Critical Illness | 367 | 0.73 |
| 妇幼 | Maternal and Child Health | 796 | 1.55 | 卫生 | Health | 356 | 0.71 |
| 健康 | Health | 709 | 1.38 | 提供 | Provide | 319 | 0.69 |
| 建设 | Construction | 595 | 1.16 | 落实 | Implementation | 261 | 0.62 |
| 加强 | Strengthen | 537 | 1.04 | 评估 | Evaluation | 257 | 0.51 |
| 管理 | Management | 512 | 1.00 | 新生儿 | Newborn | 250 | 0.50 |
| 开展 | Carry Out | 476 | 0.93 | 提升 | Improve | 246 | 0.49 |
| 产科 | Obstetrics | 454 | 0.88 | 友好 | Friendliness | 234 | 0.49 |
