# Supplementary material for: A quantitative content analysis of Chinese policies on fertility-friendly hospital development: a three-dimensional framework
Source: Front Public Health. 2026 Sep 4;14:1953541. doi: 10.3389/fpubh.2026.1953541 (PMC13585940; doi:10.3389/fpubh.2026.1953541)
Supplement: Supplementary file 1 [file Data_sheet_1.zip › Supplementary Material/Supplementary Table S1. Complete corpus of policy documents included in the analysis.docx]

**Supplementary Table S2. Complete corpus of policy documents included in the analysis**

| **No.** | **Policy Name** | **Issuing Authority** | **Issue Date** | **Administrative Level** | **Document Type** | **URL** |
| --- | --- | --- | --- | --- | --- | --- |
| 01 | Notice on Issuing the Implementation Plan for the Healthcare Capacity Strengthening Project in the Inner Mongolia Autonomous Region | Health Commission of the Inner Mongolia Autonomous Region | 2026-01-15 | Provincial | Notice | https://wjw.nmg.gov.cn/zzb/wjtz/202601/t20260120_2851010.html |
| 02 | Opinions on Promoting the Development of Fertility-Friendly Hospitals | General Office of the National Health Commission; General Office of the National Development and Reform Commission; General Office of the Ministry of Finance; General Office of the National Healthcare Security Administration | 2024-12-24 | National | Opinions | https://www.gov.cn/zhengce/zhengceku/202501/content_6999132.htm |
| 03 | Notice on Strengthening the Management of Midwifery Services | General Office of the National Health Commission | 2024-03-16 | National | Notice | https://www.gov.cn/zhengce/zhengceku/202403/content_6942014.htm |
| 04 | Guiding Opinions on Further Improving and Implementing Measures to Support Proactive Childbearing | National Health Commission and 16 other departments | 2022-07-25 | National | Opinions | http://www.gov.cn/zhengce/zhengceku/2022-08/16/content_5705882.htm |
| 05 | Notice on Issuing the Action Plan for Enhancing Maternal and Infant Safety (2021–2025) | National Health Commission | 2021-10-09 | National | Notice | http://www.gov.cn/zhengce/zhengceku/2021-10/15/content_5642740.htm |
| 06 | Notice of the General Office of the State Council on Issuing Several Measures to Accelerate the Improvement of the Fertility Support Policy System and Promote the Development of a Fertility-Friendly Society | General Office of the State Council | 2024-10-19 | National | Notice | https://www.gov.cn/zhengce/zhengceku/202410/content_6983486.htm |
| 07 | Notice of the State Council on Issuing the Outline for Women's Development in China and the Outline for Children's Development in China | State Council | 2021-09-08 | National | Notice | http://www.gov.cn/zhengce/zhengceku/2021-09/27/content_5639412.htm |
| 08 | Notice on Comprehensively Promoting Labor Analgesia | National Health Commission; National Administration of Traditional Chinese Medicine; National Disease Control and Prevention Administration | 2025-05-19 | National | Notice | https://www.gov.cn/zhengce/zhengceku/202506/content_7026664.htm |
| 09 | Notice of the General Office of Beijing Municipal People’s Government on Issuing the Beijing Work Measures for Improving the Fertility Support Policy System and Promoting the Development of a Fertility-Friendly Society | General Office of Beijing Municipal People’s Government | 2025-07-23 | Provincial | Notice | https://www.beijing.gov.cn/zhengce/zhengcefagui/202507/t20250731_4162855.html |
| 10 | Notice of Beijing Municipal Medical Insurance Bureau on Issuing the Key Tasks for Beijing Medical Security in 2025 | Beijing Municipal Medical Insurance Bureau | 2025-03-28 | Provincial | Notice | https://ybj.beijing.gov.cn/zwgk/2024zcwj/202504/t20250414_4063938.html |
| 11 | Implementation Opinions on Developing Fertility-Friendly Hospitals and Child-Friendly Hospitals | Beijing Municipal Commission of Development and Reform | 2025-08-15 | Provincial | Opinions | https://wjw.beijing.gov.cn/zwgk_20040/zcwj2024/202510/t20251027_4241020.html |
| 12 | Notice of the CPC Beijing Municipal Committee and Beijing Municipal People’s Government on Issuing the Implementation Plan for Optimizing Fertility Policy and Promoting Long-Term and Balanced Population Development | CPC Beijing Municipal Committee | 2022-01-21 | Provincial | Notice | https://www.beijing.gov.cn/zhengce/zhengcefagui/202202/t20220209_2606802.html |
| 13 | Notice of Beijing Municipal People’s Government on Issuing the Beijing 14th Five-Year Plan for the Development of Women and Children | Beijing Municipal People’s Government | 2021-11-24 | Provincial | Notice | https://www.beijing.gov.cn/zhengce/zhengcefagui/202112/t20211215_2561570.html |
| 14 | Implementation Opinions on Strengthening Basic Medical and Healthcare Services Throughout the Reproductive Process | Beijing Municipal Health Commission | 2018-12-28 | Provincial | Opinions | https://wjw.beijing.gov.cn/zwgk_20040/zcwj2024/202405/t20240514_3681532.html |
| 15 | Notice of Chongqing Municipal People’s Government on Issuing the Chongqing Women’s Development Plan (2021–2030) and Chongqing Children’s Development Plan (2021–2030) | Chongqing Municipal People’s Government | 2022-07-29 | Provincial | Notice | http://www.cq.gov.cn/zwgk/zfxxgkml/szfwj/qtgw/202208/t20220812_11000590.html |
| 16 | Notice of the General Office of Zhejiang Provincial People’s Government on Issuing Several Measures to Improve the Fertility Support Policy System | General Office of Zhejiang Provincial People’s Government | 2025-02-19 | Provincial | Notice | https://zfgjj.wenzhou.gov.cn/art/2025/4/27/art_1229129105_2038946.html |
| 17 | Notice of the General Office of Fujian Provincial People’s Government on Issuing Several Measures to Promote the Development of a Fertility-Friendly Society | General Office of Fujian Provincial People’s Government | 2025-08-07 | Provincial | Notice | http://www.fujian.gov.cn/zwgk/zxwj/szfbgtwj/202508/t20250812_6990289.htm |
| 18 | Notice of the People’s Government of Guangxi Zhuang Autonomous Region on Issuing the 14th Five-Year Plan for Health Development in Guangxi | People’s Government of Guangxi Zhuang Autonomous Region | 2022-06-13 | Provincial | Notice | http://www.gxzf.gov.cn/zfwj/zzqrmzfwj_34845/t12039703.shtml |
| 19 | Implementation Plan of the CPC Fujian Provincial Committee and Fujian Provincial People’s Government for Optimizing Fertility Policy and Promoting Long-Term and Balanced Population Development | Fujian Provincial People’s Government | 2022-05-27 | Provincial | Notice | http://www.fujian.gov.cn/zwgk/zxwj/szfwj/202205/t20220527_5920427.htm |
| 20 | Notice of the People’s Government of Guangxi Zhuang Autonomous Region on Issuing the Guangxi Women’s Development Plan and Guangxi Children’s Development Plan | People’s Government of Guangxi Zhuang Autonomous Region | 2021-12-18 | Provincial | Notice | http://www.gxzf.gov.cn/zfwj/zzqrmzfwj_34845/t11011207.shtml |
| 21 | Notice of the General Office of Guizhou Provincial People’s Government on Issuing Several Measures to Accelerate the Improvement of the Fertility Support Policy System and Promote the Development of a Fertility-Friendly Society | General Office of Guizhou Provincial People’s Government | 2025-12-16 | Provincial | Notice | https://www.guizhou.gov.cn/zwgk/zcfg/szfwj/qfbf/202512/t20251230_89104861.html |
| 22 | Notice of Guizhou Provincial People’s Government on Issuing the Guizhou Women’s Development Plan and Guizhou Children’s Development Plan | Guizhou Provincial People’s Government | 2021-12-27 | Provincial | Notice | https://www.guizhou.gov.cn/zwgk/zcfg/szfwj/qff/202201/t20220121_72387746.html |
| 23 | Notice of the CPC Guizhou Provincial Committee and Guizhou Provincial People’s Government on Issuing the Implementation Plan for Optimizing Fertility Policy and Promoting Long-Term and Balanced Population Development | Guizhou Provincial People’s Government | 2021-12-19 | Provincial | Notice | https://www.guizhou.gov.cn/zwgk/zcfg/swygwj/202112/t20211227_72137104.html |
| 24 | Notice of Guizhou Provincial Health Commission on Issuing the Implementation Plan for the 2021 Maternal and Child Healthcare Institution Capacity-Building Project | Guizhou Provincial Health Commission | 2021-07-06 | Provincial | Notice | https://wjw.guizhou.gov.cn/zwgk/jcxxgk/zcwj/bmwj/202109/t20210913_78925161.html |
| 25 | Notice of Hainan Provincial People’s Government on Issuing the Hainan Women’s Development Plan (2021–2030) and Hainan Children’s Development Plan (2021–2030) | Hainan Provincial People’s Government | 2022-09-15 | Provincial | Notice | http://www.hainan.gov.cn/hainan/szfwj/202209/e7a478ec1c40412db262e05df39c499b.shtml |
| 26 | Notice of Hebei Provincial People’s Government on Issuing the Hebei Women’s Development Plan (2021–2030) and Hebei Children’s Development Plan (2021–2030) | Hebei Provincial People’s Government | 2021-12-22 | Provincial | Notice | https://www.hebei.gov.cn/columns/b1b59c8c-81a3-4cf2-b876-8618919c0049/202308/14/a55ab098-10d9-41c7-a5ba-9fc967bd26f9.html |
| 27 | Notice of Henan Provincial People’s Government on Issuing the Henan Women’s Development Plan (2021–2030) and Henan Children’s Development Plan (2021–2030) | Henan Provincial People’s Government | 2022-03-17 | Provincial | Notice | https://www.henan.gov.cn/2022/03-22/2418814.html |
| 28 | Notice of Heilongjiang Provincial People’s Government on Issuing the Heilongjiang Women’s Development Plan (2021–2025) and Heilongjiang Children’s Development Plan (2021–2025) | Heilongjiang Provincial People’s Government | 2021-12-27 | Provincial | Notice | https://www.hlj.gov.cn/hlj/c108372/202112/c00_31181955.shtml |
| 29 | Notice of Hubei Provincial People’s Government on Issuing the Hubei Women’s Development Plan (2021–2030) and Hubei Children’s Development Plan (2021–2030) | Hubei Provincial People’s Government | 2021-12-25 | Provincial | Notice | http://www.hubei.gov.cn/zfwj/ezf/202112/t20211231_3947154.shtml |
| 30 | Notice of Qinghai Provincial People’s Government on Issuing the Qinghai Women’s Development Plan and Qinghai Children’s Development Plan | Qinghai Provincial People’s Government | 2022-02-11 | Provincial | Notice | http://www.qinghai.gov.cn/xxgk/xxgk/fd/zfwj/202203/t20220309_189263.html |
| 31 | Notice of the General Office of Hunan Provincial People’s Government on Issuing Ten Measures to Improve Fertility Support Policies and Promote the Development of a Fertility-Friendly Society | General Office of Hunan Provincial People’s Government | 2025-10-21 | Provincial | Notice | https://www.hunan.gov.cn/hnszf/xxgk/wjk/szfbgt/202510/t20251030_33837131.html |
| 32 | Notice on Issuing the Implementation Opinions of Jilin Province on Further Improving and Implementing Measures to Support Proactive Childbearing | Jilin Provincial Health Commission | 2023-12-29 | Provincial | Notice | http://xxgk.jl.gov.cn/zcbm/fgw_98077/xxgkmlqy/202401/t20240104_8860144.html |
| 33 | Notice of the General Office of Jiangsu Provincial People’s Government on Issuing Several Measures to Accelerate the Improvement of the Fertility Support Policy System and Promote the Development of a Fertility-Friendly Society | Jiangsu Provincial People’s Government | 2025-01-11 | Provincial | Notice | http://www.jiangsu.gov.cn/art/2025/2/20/art_46144_11496032.html |
| 34 | Notice of the General Office of Jiangsu Provincial People’s Government on Issuing the Implementation Plan for Further Improving the Medical and Health Service System | Jiangsu Provincial People’s Government | 2024-01-05 | Provincial | Notice | http://www.jiangsu.gov.cn/art/2024/1/31/art_46144_11141650.html |
| 35 | Notice of the General Office of Jiangsu Provincial People’s Government on Issuing the Jiangsu Women’s Development Plan and Children’s Development Plan | Jiangsu Provincial People’s Government | 2021-12-02 | Provincial | Notice | http://www.jiangsu.gov.cn/art/2021/12/14/art_46144_10191305.html |
| 36 | Notice of the General Office of Jiangsu Provincial People’s Government on Issuing the Jiangsu 14th Five-Year Plan for Health Development | Jiangsu Provincial People’s Government | 2021-09-30 | Provincial | Notice | http://www.jiangsu.gov.cn/art/2021/10/15/art_46144_10075225.html |
| 37 | Notice of the General Office of Jiangxi Provincial People’s Government on Issuing Several Measures of Jiangxi Province to Accelerate the Improvement of the Fertility Support Policy System | General Office of Jiangxi Provincial People’s Government | 2025-07-10 | Provincial | Notice | https://www.jiangxi.gov.cn/jxsrmzf/szfbg100/pc/content/content_1944563193291153408.html |
| 38 | Notice on Implementing the Requirements of the Two Documents on Promoting the Development of Fertility-Friendly Hospitals and Child-Friendly Hospitals | Jiangxi Provincial Health Commission | 2025-06-10 | Provincial | Notice | https://hc.jiangxi.gov.cn/jxswsjkwyh/zcwj121/pc/content/content_1936226759108841472.html |
| 39 | Notice of the Health Commission of the Inner Mongolia Autonomous Region and 15 Other Departments on Issuing Several Measures of the Inner Mongolia Autonomous Region to Improve and Implement Proactive Fertility Support | Health Commission of the Inner Mongolia Autonomous Region | 2022-12-20 | Provincial | Notice | https://wjw.nmg.gov.cn/zzb/gfxwj/202301/t20230116_2215589.html |
| 40 | Notice of the General Office of Shanghai Municipal People’s Government on Issuing Several Measures to Accelerate the Improvement of the Fertility Support Policy System and Promote the Development of a Fertility-Friendly Society | General Office of Shanghai Municipal People’s Government | 2024-12-12 | Provincial | Notice | https://www.shanghai.gov.cn/gwk/search/content/fab6bad7a4ec42a3bf7566c4782ec1fd |
| 41 | Notice of the People’s Government of the Inner Mongolia Autonomous Region on Issuing the Women’s Development Outline (2021–2030) and Children’s Development Outline (2021–2030) | People’s Government of the Inner Mongolia Autonomous Region | 2021-12-20 | Provincial | Notice | https://www.nmg.gov.cn/zwgk/zfxxgk/zfxxgkml/ghxx/zxgh/202112/t20211229_1987238.html |
| 42 | Notice of the General Office of the People’s Government of the Inner Mongolia Autonomous Region on Issuing the 14th Five-Year Plan for Health Development of the Autonomous Region | General Office of the People’s Government of the Inner Mongolia Autonomous Region | 2021-10-16 | Provincial | Notice | https://www.nmg.gov.cn/zwgk/zfxxgk/zfxxgkml/ghxx/zxgh/202110/t20211026_1915925.html |
| 43 | Notice of the General Office of the Health Commission of the Inner Mongolia Autonomous Region on Issuing the 2020 Maternal and Infant Safety and Healthy Children Action Plan | General Office of the Health Commission of the Inner Mongolia Autonomous Region | 2020-03-11 | Provincial | Notice | http://wjw.nmg.gov.cn/zfxxgk/fdzzgknr/wjzt/202106/t20210615_1620985.html |
| 44 | Notice of the General Office of the People’s Government of the Ningxia Hui Autonomous Region on Issuing the Women’s and Children’s Development Plans of the Ningxia Hui Autonomous Region | General Office of the People’s Government of the Ningxia Hui Autonomous Region | 2021-12-29 | Provincial | Notice | https://www.nx.gov.cn/zwgk/qzfwj/202201/t20220113_3282662.html |
| 45 | Notice of the General Office of the People’s Government of the Ningxia Hui Autonomous Region on Issuing Several Measures to Accelerate the Improvement of the Fertility Support Policy System and Promote the Development of a Fertility-Friendly Society | General Office of the People’s Government of the Ningxia Hui Autonomous Region | 2025-07-24 | Provincial | Notice | https://www.nx.gov.cn/zwgk/qzfwj/202508/t20250804_4980171.html |
| 46 | Notice of Shandong Provincial People’s Government on Issuing the Shandong 14th Five-Year Women’s Development Plan and the Shandong 14th Five-Year Children’s Development Plan | Shandong Provincial People’s Government | 2021-10-15 | Provincial | Notice | http://www.shandong.gov.cn/jpaas-jpolicy-web-server/front/info/detail?iid=b4e665fff91049abba8ae3e15195f053 |
| 47 | Notice of the CPC Shanxi Provincial Committee and Shanxi Provincial People’s Government on Issuing the Implementation Plan for Optimizing Fertility Policy and Promoting Long-Term and Balanced Population Development | CPC Shanxi Provincial Committee and Shanxi Provincial People’s Government | 2022-09-30 | Provincial | Notice | http://www.shanxi.gov.cn/zcwjk/swwj/202504/t20250410_9806351.shtml |
| 48 | Notice on Issuing the Implementation Plan for the Maternal and Infant Safety Enhancement Action Plan (2022–2025) and the Implementation Plan for the Healthy Children Enhancement Action Plan (2022–2025) | Shaanxi Provincial Health Commission | 2022-04-15 | Provincial | Notice | https://sxwjw.shaanxi.gov.cn/ywgz/fyjk/gzdt_922/202206/t20220616_2224881.html |
| 49 | Notice of the General Office of Shaanxi Provincial People’s Government on Issuing the Women’s Development Plan and Children’s Development Plan | General Office of Shaanxi Provincial People’s Government | 2021-12-13 | Provincial | Notice | https://www.shaanxi.gov.cn/zfxxgk/fdzdgknr/zcwj/nszfbgtwj/szbf/202208/t20220808_2235768.html |
| 50 | Notice of the General Office of Yunnan Provincial People’s Government on Issuing Fifteen Measures to Accelerate the Improvement of the Fertility Support Policy System and Promote the Development of a Fertility-Friendly Society | General Office of Yunnan Provincial People’s Government | 2025-12-14 | Provincial | Notice | https://ynswsjkw.yn.gov.cn/html/2025/qitawenjianxin_1217/4024187.html |
| 51 | Notice of Yunnan Provincial People’s Government on Issuing the Yunnan Women’s Development Plan and Yunnan Children’s Development Plan | Yunnan Provincial People’s Government | 2022-05-31 | Provincial | Notice | https://www.yn.gov.cn/zwgk/zcwj/zxwj/202206/t20220610_243046.html |
| 52 | Notice on Strengthening Maternal Health Care under the Universal Two-Child Policy | Beijing Municipal Commission of Health and Family Planning | 2016-04-07 | Provincial | Notice | https://wjw.beijing.gov.cn/zwgk_20040/zcwj2024/202405/t20240515_3683284.html |
