# Supplementary material for: A quantitative content analysis of Chinese policies on fertility-friendly hospital development: a three-dimensional framework
Source: Front Public Health. 2026 Sep 4;14:1953541. doi: 10.3389/fpubh.2026.1953541 (PMC13585940; doi:10.3389/fpubh.2026.1953541)
Supplement: Supplementary file 1 [file Data_sheet_1.zip › Supplementary Material/Supplementary Table S2. Coding framework.docx]

**Supplementary Table S3. Complete coding framework, operational definitions, decision rules, and representative coding examples**

| **Dimension** | **Category / Node** | **Operational definition** | **Decision rule** | **Representative coding example** | **Coding rationale** |
| --- | --- | --- | --- | --- | --- |
| X-dimension: Policy tools Supply-side | **Financial Support** | Provides financial support for fertility-friendly hospital development through government funding, earmarked funds, and construction subsidies. | Code when the policy explicitly allocates, guarantees, or increases government fiscal expenditure, earmarked funds, operating funds, construction subsidies, or other direct financial inputs. | 各区要加大对妇幼健康事业的经常性及专项投入，确保辖区妇幼健康发展与经济社会发展水平相适应。结合国家对妇幼保健公共卫生人员配置要求，切实保障妇幼保健机构提供公共卫生服务所需人员、公用经费以及组织管理、监督考核等业务经费。  *English translation:* Districts should increase recurrent and earmarked investment in maternal and child health services and ensure adequate funding for personnel, operating expenses, organizational management, supervision, and performance assessment required for the provision of public health services by maternal and child health institutions. | The segment explicitly requires recurrent and earmarked fiscal investment and therefore represents direct financial support. |
| X-dimension: Policy tools Supply-side | **Infrastructure and Equipment Development** | Improves inpatient and childbirth conditions for pregnant and postpartum women by increasing obstetric bed capacity, renovating ward environments, and upgrading facilities and equipment. | Code when the policy expands, constructs, renovates, or reallocates physical facilities, beds, service spaces, or medical equipment. If the text primarily describes healthcare service delivery, code as Service Provision instead. | 通过盘活存量、筹划增量，合理规划医院内部床位设置，通过调整妇产科内部、科室之间床位资源，增加产科床位供给。  *English translation:* Existing and additional resources should be optimized, hospital bed allocation should be rationally planned, and obstetric bed capacity should be increased by reallocating beds within and across departments. | The principal policy measure is the expansion and reallocation of physical obstetric capacity. |
| X-dimension: Policy tools Supply-side | **Health Workforce Development** | Enhances the capacity to provide fertility-related healthcare services by training and recruiting professionals in understaffed specialties, such as obstetrics and midwifery, and providing performance-based incentives. | Code when the policy concerns staffing levels, recruitment, training, professional-title promotion, career development, remuneration incentives, or retention of healthcare professionals. | 各助产机构要保证助产人员配置，三级、二级助产机构产科医师不少于10人、8人，产房助产士不少于8人、6人；年分娩量超过1000人、800人的助产机构，分娩量每增加1000人，至少增加产科医生和助产士各3人。  *English translation:* Maternity care institutions should ensure adequate maternity-care staffing. Tertiary and secondary maternity care institutions should have no fewer than 10 and 8 obstetricians and no fewer than 8 and 6 delivery-room midwives, respectively. Additional obstetricians and midwives should be added according to annual delivery volume. | The segment specifies minimum staffing levels and workforce expansion requirements. |
| X-dimension: Policy tools Supply-side | **Service Provision** | Provides women with a continuum of fertility-related healthcare services spanning preconception, antenatal care, childbirth, and postpartum care. | Code when the policy directly requires the provision, expansion, standardization, or improvement of specific fertility-related healthcare services. | 提供更加规范、安全、优质的辅助生殖技术服务。  *English translation:* Provide more standardized, safe, and high-quality assisted reproductive technology services. | The segment directly requires the provision and improvement of a specific fertility-related healthcare service. |
| X-dimension: Policy tools Supply-side | **Digital Empowerment** | Improves the efficiency and patient experience of obstetric services through digital technologies, including online appointment systems, telemedicine, internet-based healthcare, and artificial intelligence. | Code when digital platforms, information systems, telemedicine, internet-based healthcare, artificial intelligence, or similar technologies are explicitly used to support care delivery or management. | 强化妇幼健康信息科技支撑，打造生命周期“互联网+妇幼健康”服务，推广“云上妇幼”。  *English translation:* Strengthen information-technology support for maternal and child health, develop life-course “Internet Plus Maternal and Child Health” services, and promote cloud-based maternal and child healthcare. | The segment explicitly uses internet-based and cloud technologies to support maternal and child health services. |
| X-dimension: Policy tools Demand-side | **Pricing and Payment Support** | Reduces out-of-pocket costs for fertility-related healthcare among disadvantaged groups through government subsidies, pricing adjustments, and financial assistance mechanisms. | Code when the policy directly addresses healthcare service prices, price-setting mechanisms, dynamic price adjustment, payment standards, subsidies, or financial assistance. Insurance coverage itself is coded separately. | 医保部门加快落实《产科类医疗服务价格项目立项指南（试行）》，合理确定产科价格水平，促进产科平稳运行和高质量发展。在确保医保基金运行安全、符合调价启动条件的前提下，将产科、儿科（新生儿科）医疗服务价格纳入动态调整机制中统筹考虑。  *English translation:* Healthcare security authorities should accelerate implementation of the Guidelines for Establishing Obstetric Medical Service Pricing Items (Trial), set appropriate prices for obstetric services, and incorporate obstetric and pediatric (neonatal) medical service prices into the dynamic price-adjustment mechanism when relevant conditions are met. | The segment focuses on setting and dynamically adjusting medical service prices rather than insurance reimbursement. |
| X-dimension: Policy tools Demand-side | **Health Insurance Coverage** | Reduces the financial burden of fertility-related healthcare by expanding health insurance coverage for eligible services. | Code when the policy explicitly refers to inclusion in maternity or basic medical insurance, reimbursement coverage, or insurance payment. | 将符合条件的产前检查、分娩等生育医疗费用纳入生育保险或由基本医疗保险按规定予以报销。  *English translation:* Eligible medical expenses for antenatal examinations, childbirth, and other maternity care services should be covered by maternity insurance or reimbursed through basic medical insurance in accordance with relevant regulations. | The segment explicitly concerns insurance coverage and reimbursement of maternity-related healthcare costs. |
| X-dimension: Policy tools Demand-side | **Patient-Convenience Services** | Improves access to healthcare by providing patient-centered services, such as advance scheduling for hospital admission and childbirth, one-stop administrative processing, and real-time settlement of medical expenses. | Code when the primary purpose is to make healthcare services easier or more convenient to access and use, including appointments, self-service facilities, simplified administrative procedures, or one-stop services. | 完善自助服务设备，提供便民设施，开展预约诊疗服务，改善孕产妇、儿童就诊体验。  *English translation:* Improve self-service equipment, provide patient-convenience facilities, offer appointment-based medical services, and improve the healthcare experience of pregnant and postpartum women and children. | The measures are explicitly intended to improve convenience and the patient experience. |
| X-dimension: Policy tools Demand-side | **Equity and Rights Protection** | Ensures that all population groups have equitable access to fertility-related healthcare and that their reproductive healthcare rights are protected. | Code when the policy explicitly addresses informed consent, voluntary choice, reproductive rights, equitable access, or protection of vulnerable groups. Insurance reimbursement alone is not sufficient unless equity or rights protection is explicitly addressed. | 认真履行告知义务并取得知情同意，方便患者充分了解和自愿选择分娩镇痛服务。  *English translation:* Healthcare providers should fulfill their obligation to provide adequate information and obtain informed consent, enabling patients to fully understand and voluntarily choose labor analgesia services. | The segment explicitly safeguards informed consent and voluntary choice. |
| X-dimension: Policy tools Environmental | **Target Setting and Planning** | Sets phased and measurable targets and specifies the implementation pathways and timelines for developing fertility-friendly hospitals across different levels of maternity care institutions. | Code when the policy specifies quantitative targets, coverage rates, deadlines, implementation stages, or planned development objectives. | 到2025年底，全省15%的助产机构达到生育友好医院建设标准，重点覆盖三级综合医院、妇幼保健院；全省15%的妇幼保健院设置“优生优育指导中心”。  *English translation:* By the end of 2025, 15% of maternity care institutions in the province should meet the standards for fertility-friendly hospitals, with priority coverage of tertiary general hospitals and maternal and child health hospitals; 15% of maternal and child health hospitals should establish preconception and reproductive health guidance centers. | The segment specifies a clear deadline, quantitative coverage target, and development objective. |
| X-dimension: Policy tools Environmental | **Advocacy of Fertility-Friendly Principles** | Promotes broader social recognition of fertility-friendly principles and fosters a supportive social environment. | Code when the policy emphasizes publicity, education, communication, dissemination, advocacy, or public awareness. | 各区要充分利用电视、广播、新媒体等形式，开展孕产期保健知识系列宣传和本区孕妇建册、建档、转诊服务内容和流程宣传，引导群众合理、就近选择分娩地点。  *English translation:* Districts should use television, radio, new media, and other channels to disseminate maternal healthcare knowledge and information on maternal registration, health-record establishment, and referral procedures, and to guide the public in choosing appropriate nearby places for childbirth. | The primary policy instrument is public communication and behavioral guidance. |
| X-dimension: Policy tools Environmental | **Standards Development** | Provides technical guidance for fertility-friendly hospital development by establishing construction guidelines, service specifications, and clinical standards. | Code when the policy establishes or strengthens formal standards, technical specifications, management systems, operating requirements, or quality criteria. | 各助产机构应根据自身功能、任务、规模，配备与输血工作相适应的专业技术人员、设施、设备，根据有关规定和用血需求设置输血科或血库，并向机构的登记部门申请执业登记。建立并完善产科用血应急预案等临床用血管理制度和规范，开展产科用血质量评价，保障产科用血医疗安全。  *English translation:* Maternity care institutions should allocate professional personnel, facilities, and equipment appropriate to their transfusion-related functions, establish a transfusion department or blood bank as required, and develop clinical blood-use management systems and standards, including emergency obstetric blood-use plans and quality evaluation mechanisms. | The segment establishes explicit institutional, technical, and management requirements and therefore represents standards development. |
| X-dimension: Policy tools Environmental | **Organizational Coordination** | Establishes a coordinated maternal safety system through multidisciplinary collaboration, consultation and referral mechanisms, and regional emergency care networks. | Code when the policy emphasizes departmental coordination, institutional collaboration, multidisciplinary mechanisms, referral networks, or joint implementation. | 各地各有关部门要强化部门协同，加大“两个友好医院”建设投入力度，卫生健康、教育、医保、妇联等部门要完善相关配套支持措施，发展改革、财政部门要落实资金投入。  *English translation:* Relevant local departments should strengthen interdepartmental coordination. Health, education, healthcare security, and women’s federation authorities should improve supporting measures, while development and reform and finance authorities should ensure the required funding. | The segment explicitly assigns complementary responsibilities to multiple departments and therefore reflects organizational coordination. |
| X-dimension: Policy tools Environmental | **Monitoring and Performance Evaluation** | Establishes a closed-loop management system through the development of evaluation criteria, satisfaction surveys, and tiered accreditation and review. | Code when the policy concerns monitoring, supervision, assessment, inspection, reporting, feedback, accreditation, accountability, rewards, or penalties. | 全面开展孕产妇危重症评审。完善孕产妇死亡督促检查、个案报告、现场调查及通报约谈制度，严格奖惩。  *English translation:* Comprehensive reviews of severe maternal cases should be conducted, and systems for maternal-death supervision, case reporting, field investigation, notification, and accountability interviews should be improved, with strict rewards and penalties. | The segment establishes explicit mechanisms for review, supervision, reporting, and accountability. |
| Y-dimension: Stakeholders | **Government agencies** | Government bodies responsible for policy formulation, implementation, coordination, funding, regulation, and oversight. | Code when a government department is explicitly assigned responsibility for policy formulation, coordination, supervision, financial support, or administrative implementation. | 各区卫生计生委要加强属地管理，对辖区助产机构逐一开展严格督导。  *English translation:* District health and family planning commissions should strengthen territorial management and conduct rigorous supervision of maternity care institutions within their jurisdictions. | The government health authority is explicitly identified as the actor responsible for supervision and administrative management. |
| Y-dimension: Stakeholders | **Maternity care institutions** | General hospitals, traditional Chinese medicine hospitals, maternal and child health hospitals, obstetrics and gynecology hospitals, and other institutions responsible for implementing fertility-friendly initiatives and delivering related services. | Code when maternity-related medical institutions are explicitly responsible for service delivery, clinical management, organizational capacity development, or implementation. | 各助产机构要落实产科质量管理办公室职责，对门诊接收的具有严重合并症、极严重高危因素和不宜妊娠孕妇组织多学科专家会诊，出具高危孕妇风险评估书面建议，制定后续治疗方案。  *English translation:* Maternity care institutions should fulfill the responsibilities of their obstetric quality management offices, organize multidisciplinary consultations for women with severe comorbidities or extremely high-risk conditions, provide written risk-assessment recommendations, and develop subsequent treatment plans. | Maternity care institutions are explicitly assigned responsibility for clinical assessment and management. |
| Y-dimension: Stakeholders | **Healthcare professionals** | Professionals in obstetrics, neonatology, nutrition, midwifery, nursing, and related disciplines who provide fertility-related healthcare. | Code when individual healthcare professionals or professional groups are explicitly targeted for staffing, recruitment, training, promotion, incentives, or care delivery. | 各助产机构要增加每年新入职人员中产科医生、助产士和护士比例。在同级别人员职称晋升中，优先产科人员职称晋升，增强产科人才吸引力。  *English translation:* Maternity care institutions should increase the proportion of newly recruited obstetricians, midwives, and nurses and give priority to obstetric personnel in professional-title promotion to enhance the attractiveness of obstetric careers. | The segment explicitly targets healthcare professionals in relation to recruitment and career development. |
| Y-dimension: Stakeholders | **Women and families of reproductive age** | Women of reproductive age, pregnant and postpartum women, newborns, and their families as recipients, beneficiaries, and rights holders of fertility-related policies and services. | Code when these populations are explicitly identified as beneficiaries, service recipients, target populations, or rights holders. | 将生殖健康服务融入妇女健康管理全过程，保障妇女享有避孕节育的知情权和选择权，预防非意愿妊娠。  *English translation:* Reproductive health services should be integrated throughout women’s health management, while safeguarding women’s rights to informed decision-making and choice regarding contraception and preventing unintended pregnancies. | Women are explicitly identified as beneficiaries and rights holders. |
| Y-dimension: Stakeholders | **Social organizations** | Social organizations and other non-governmental actors that support fertility-related services or participate in collaborative implementation. | Code only when the surrounding policy context explicitly identifies a social organization or related non-governmental actor as the responsible participant. | 按照职责积极支持生育服务发展。  *English translation:* Actively support the development of fertility-related services in accordance with assigned responsibilities. | The segment was coded as Social organizations because the surrounding policy context explicitly identifies the relevant social organization as the actor. |
| Y-dimension: Stakeholders | **Primary healthcare institutions** | Community health service centers, township health centers, and other primary-level healthcare institutions supporting maternal healthcare and continuity of care. | Code when primary healthcare institutions are explicitly responsible for health-record establishment, community management, follow-up, referral, or continuity of maternal healthcare. | 各社区卫生服务机构应合理配置妇幼保健人员，保证孕妇在每个工作日内能够建立《母子健康档案》，杜绝择日建册。有条件的机构可探索建册预约机制。  *English translation:* Community health service institutions should appropriately allocate maternal and child health personnel and ensure that pregnant women can establish a Maternal and Child Health Record on any working day. Institutions with appropriate conditions may explore appointment-based record establishment. | Community health service institutions are explicitly identified as the actors responsible for primary maternal healthcare services. |
| Z-dimension: Policy objectives | **Institutional friendliness** | Refers to policy objectives aimed at improving institutional arrangements, governance mechanisms, organizational capacity, staffing systems, and supporting mechanisms for fertility-friendly hospital development. | Code when the primary intended outcome concerns institutional systems, organizational mechanisms, staffing arrangements, remuneration, incentives, or governance capacity. | 各助产机构在产科薪酬分配等方面要加大政策倾斜力度，科学设定产科绩效工资总额及增长机制，在薪酬分配上充分体现产科工作风险及落实国家生育政策调整等因素，制定产科人员薪酬倾斜方案，原则上产科工作人员绩效工资应高于本单位同级别工作人员的平均水平。  *English translation:* Maternity care institutions should strengthen policy support for obstetric remuneration, establish appropriate mechanisms for the total amount and growth of performance-based pay, and ensure that compensation reflects occupational risks in obstetric work and adjustments to national fertility policies. In principle, the performance-based pay of obstetric staff should be higher than the average level of staff at the same grade within the institution. | The intended outcome is improvement in institutional remuneration and incentive mechanisms. |
| Z-dimension: Policy objectives | **Environmental friendliness** | Refers to policy objectives aimed at improving the physical and service environment to create a more comfortable, convenient, and supportive care setting. | Code when the intended outcome primarily concerns physical space, facilities, environmental accessibility, privacy, comfort, or supportive amenities. | 在用人单位和医院、商场、旅游景点等公共场所普遍设立母婴室。  *English translation:* Mother-and-baby rooms should be widely established in workplaces, hospitals, shopping centers, tourist attractions, and other public places. | The intended outcome is improvement of the physical and supportive environment for mothers and infants. |
| Z-dimension: Policy objectives | **Continuum-of-care friendliness** | Refers to policy objectives aimed at strengthening continuity and integration of fertility-related healthcare across preconception, pregnancy, childbirth, postpartum, and related stages. | Code when the intended outcome emphasizes continuous, integrated, or coordinated healthcare across multiple stages of reproductive and maternal care. | 提供生育全程基本医疗保健服务，健全综合预警网络，实施孕产妇安全风险预防、预警、应急保障。  *English translation:* Provide basic healthcare services throughout the reproductive and maternity care continuum, strengthen comprehensive early-warning networks, and implement risk prevention, early warning, and emergency safeguards for maternal safety. | The explicit emphasis on healthcare throughout the reproductive and maternity care continuum indicates continuity across stages. |
| Z-dimension: Policy objectives | **Service model friendliness** | Refers to policy objectives aimed at developing more people-centered, diversified, supportive, and responsive models of fertility-related healthcare. | Code when the intended outcome concerns supportive or individualized service models addressing broader nutritional, psychological, behavioral, or health-management needs rather than a specific clinical workflow. | 各助产机构要创造条件设置孕期营养门诊，通过营养门诊指导，帮助孕产妇掌握科学饮食和运动，合理控制血糖、体重等，促进膳食平衡，减少巨大儿发生，促进自然分娩。  *English translation:* Maternity care institutions should establish antenatal nutrition clinics to provide guidance on appropriate diet and physical activity, help pregnant and postpartum women manage blood glucose and body weight, promote balanced nutrition, reduce the occurrence of macrosomia, and facilitate natural childbirth. | The segment establishes a specialized supportive service model addressing individualized nutritional and health-management needs. |
| Z-dimension: Policy objectives | **Clinical process friendliness** | Refers to policy objectives aimed at optimizing clinical management and care processes, including appointments, examinations, consultations, referrals, and coordination of clinical procedures. | Code when the intended outcome concerns improvement in clinical workflow, appointment procedures, examination arrangements, consultation, referral, or other care processes. | 完善产科预约诊疗制度，推进全面预约诊疗，落实分时段预约，推行检查检验集中预约。  *English translation:* Improve the appointment system for obstetric care, expand appointment-based services, implement time-specific appointments, and introduce centralized scheduling for examinations and laboratory tests. | The segment directly concerns optimization of appointment and examination procedures and therefore represents improvement in the clinical care process. |

**Note:** Coding was conducted using the smallest meaningful text segment as the basic coding unit. The same text segment could be coded across different analytical dimensions when it contained information relevant to policy tools, stakeholders, and policy objectives. Within the same dimension, multiple codes were assigned only when a text segment contained multiple independently identifiable categories. The Chinese policy segments shown in this table are representative excerpts from the included policy documents. English translations were prepared by the authors for reporting purposes. Coding decisions were based on the primary meaning expressed in each minimum meaningful text segment.
