# Supplementary material for: A quantitative content analysis of Chinese policies on fertility-friendly hospital development: a three-dimensional framework
Source: Front Public Health. 2026 Sep 4;14:1953541. doi: 10.3389/fpubh.2026.1953541 (PMC13585940; doi:10.3389/fpubh.2026.1953541)
Supplement: Supplementary file 1 [file Data_sheet_1.zip › Supplementary Material/Supplementary Table S4. Detailed distribution of policy tool categories by administrative level.docx]

## Supplementary Table S4. Detailed distribution of policy tool categories by administrative level

| Policy tool category | Subcategory | Provincial-level n (%) | National-level n (%) |
| --- | --- | --- | --- |
| Supply-side | Service Provision | 121 (28.27%) | 21 (23.86%) |
|  | Health Workforce Development | 35 (8.18%) | 6 (6.82%) |
|  | Infrastructure and Equipment Development | 48 (11.21%) | 12 (13.64%) |
|  | Digital Empowerment | 19 (4.44%) | 6 (6.82%) |
|  | Financial Support | 6 (1.40%) | 1 (1.14%) |
| Environmental | Standards Development | 16 (3.74%) | 5 (5.68%) |
|  | Monitoring and Performance Evaluation | 26 (6.07%) | 8 (9.09%) |
|  | Advocacy of Fertility-Friendly Principles | 7 (1.64%) | 3 (3.41%) |
|  | Target Setting and Planning | 16 (3.74%) | 5 (5.68%) |
|  | Organizational Coordination | 83 (19.39%) | 10 (11.36%) |
| Demand-side | Patient-Convenience Services | 9 (2.10%) | 2 (2.27%) |
|  | Pricing Support | 20 (4.67%) | 3 (3.41%) |
|  | Equity and Rights Protection | 6 (1.40%) | 3 (3.41%) |
|  | Health Insurance Coverage | 16 (3.74%) | 3 (3.41%) |

**Note:** Percentages were calculated using the total number of coded policy tool references within each administrative level as the denominator. The denominators were 428 and 88 for provincial- and national-level policy documents, respectively.
